# Unlocking the thermoelectric potential of the Ca_14_AlSb_11_ structure type

**DOI:** 10.1126/sciadv.abq3780

**Published:** 2022-09-07

**Authors:** Andrew P. Justl, Francesco Ricci, Andrew Pike, Giacomo Cerretti, Sabah K. Bux, Geoffroy Hautier, Susan M. Kauzlarich

**Affiliations:** ^1^ Department of Chemistry, University of California, One Shields Ave, Davis, CA 95616, USA.; ^2^ Institute of Condensed Matter and Nanoscience (IMCN), Université catholique de Louvain (UCLouvain), Chemin étoiles 8, bte L7.03.01, Louvain-la-Neuve 1348, Belgium.; ^3^ Thayer School of Engineering, Dartmouth College, Hanover, NH 03755, USA.; ^4^ Thermal Energy Conversion Technologies Group, Jet Propulsion Laboratory, California Institute of Technology, 4800 Oak Grove Drive, MS 277-207, Pasadena, CA 91109, USA.

## Abstract

Yb_14_MnSb_11_ and Yb_14_MgSb_11_ are among the best p-type high-temperature (>1200 K) thermoelectric materials, yet other compounds of this Ca_14_AlSb_11_ structure type have not matched their stability and efficiency. First-principles computations show that the features in the electronic structures that have been identified to lead to high thermoelectric performances are present in Yb_14_ZnSb_11_, which has been presumed to be a poor thermoelectric material. We show that the previously reported low power factor of Yb_14_ZnSb_11_ is not intrinsic and is due to the presence of a Yb_9_Zn_4+*x*_Sb_9_ impurity uniquely present in the Zn system. Phase-pure Yb_14_ZnSb_11_ synthesized through a route avoiding the impurity formation reveals its exceptional high-temperature thermoelectric properties, reaching a peak *zT* of 1.2 at 1175 K. Beyond Yb_14_ZnSb_11_, the favorable band structure features for thermoelectric performance are universal among the Ca_14_AlSb_11_ structure type, opening the possibility for high-performance thermoelectric materials.

## Teaser

The interplay of synthesis, experiments, and theory in broadening the landscape of thermoelectric materials is reported.

## INTRODUCTION

Yb_14_*M*Sb_11_ (*M* = Mn and Mg) are among the most efficient high-temperature p-type thermoelectric materials exhibiting a unitless thermoelectric figure of merit, *zT*, of 1.33 at 1275 K and 1.28 at 1175 K, respectively (*1*–*3*). The high *zT* depends on electronic and thermal transport, and a rationale for their high performance has recently emerged (*4*–*6*). The low thermal conductivity is directly linked to the complex and large crystal structure. The excellent electronic properties with high Seebeck coefficient and conductivity are linked to a multivalley Fermi surface. Both Yb_14_MnSb_11_ and Yb_14_MgSb_11_ share these attributes and show high *zT*s as a result. On the other hand, the Zn analog of the Yb_14_*M*Sb_11_ (generalized by 14-1-11) compounds, Yb_14_ZnSb_11_, has shown poor thermoelectric properties with a relatively high thermal conductivity and a low Seebeck coefficient (*7*). This is unexpected considering how similar the Zn compound is to the Mn and Mg analogs: All metals are 2+, all contain Yb and Sb, and the compounds are the same structure type. This prompts questions about how common high thermoelectric performance is within the vast space of isostructural materials forming in the Ca_14_AlSb_11_ structure type (*8*). Here, we show that contrary to a previous report (*7*), Yb_14_ZnSb_11_ has a high figure of merit (1.2 to 1.9 at 1175 to 1275 K) supported by first-principles computations, indicating similar band structures across the Zn, Mn, and Mg series. This marked improvement in figure of merit for Yb_14_ZnSb_11_ is due to a new synthetic route leading to the reduction of competing side phase impurities. Beyond Yb_14_ZnSb_11_, we show that the multivalley band structure, which is favorable to thermoelectric properties, is universal to the compounds of the Ca_14_AlSb_11_ structure type. Our work motivates the investigation of the many compositions that crystallize in the Ca_14_AlSb_11_ structure type and showcases the importance of phase purity and the development of synthetic routes to obtain high-performance thermoelectric materials.

## RESULTS AND DISCUSSION

### Band structure of Yb_14_ZnSb_11_

**Fig. 1.**
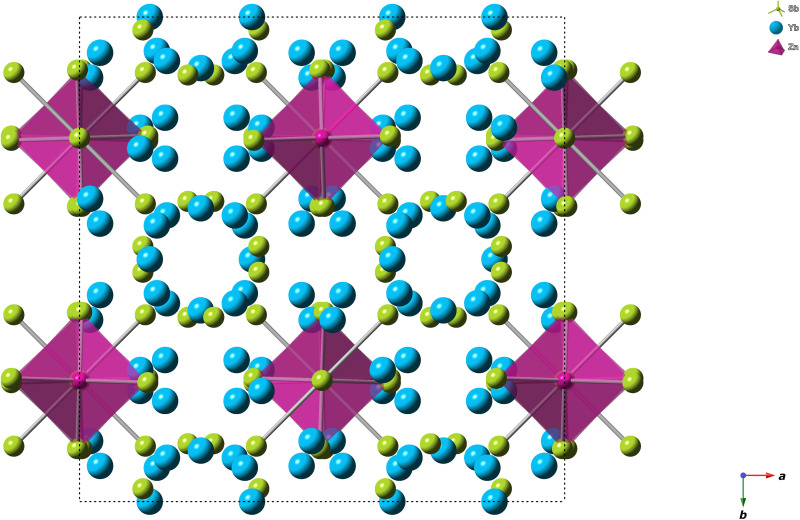
A projection view of the tetragonal (*I*4_1_/*acd*) Ca_14_AlSb_11_ unit cell down the *c* axis. The tetrahedral unit is shaded purple, and Ca is indicated in blue, Sb in green, and Al in purple.

The champion high-temperature p-type thermoelectric materials Yb_14_MnSb_11_ and Yb_14_MgSb_11_ (*zT* = 1.33 and 1.28, respectively) form in the Ca_14_AlSb_11_ structure type, which can accommodate a number of different elements (*8*). Compounds of this structure type fall under the description of Zintl phases where simple electron counting rules can be used to rationalize the bonding and are considered semiconductors (*9*–*12*). Figure 1 shows the crystal structure of Ca_14_AlSb_11_ that contains eight formula units, each of which can be described as consisting of 14 Ca^2+^ cations, a [AlSb_4_]^9−^ tetrahedron, a Sb_3_^7−^ linear unit, and four Sb^−3^ anions. The electronic properties of these materials will depend on their bonding and resultant electronic band structures.

**Fig. 2.**
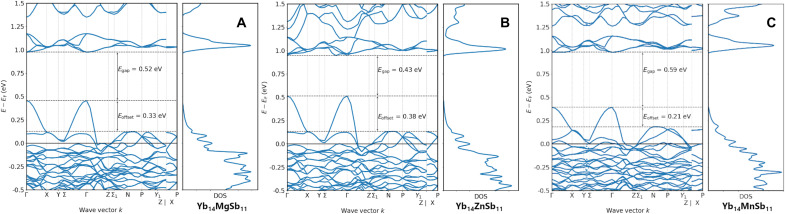
The band structures and density of states for Yb_14_*M*Sb_11_*M =* Mg, Zn, and Mn. (**A**) Yb_14_MgSb_11_, (**B**) Yb_14_ZnSb_11_, and (**C**) Yb_14_MnSb_11_ obtained from DFT.

Figure 2 shows the band structures and density of states of Yb_14_MgSb_11_, Yb_14_ZnSb_11_, and Yb_14_MnSb_11_ obtained by density functional theory (DFT). The band structures of the three compounds can be described by the multiband model recently developed for Yb_14_Mg_1−*x*_Al*_x_*Sb_11_ (*5*). In this model, the high power factor comes from the combination of a low–effective mass band present at the Γ point and a highly degenerate (*N*_v_ = 8) heavy pocket of bands between N and P. As the temperature increases, the Fermi-Dirac distribution broadens, and this pocket of degenerate, heavy bands become involved in transport because of their proximity to the Fermi level. This leads to increases in effective mass with increasing temperature and thereby large Seebeck coefficients (*5*). The three compounds show very similar band structures and therefore should all have a high power factor. A closer look at the projected density of states and an analysis of the bonding in Yb_14_ZnSb_11_ [Supplementary Materials, figs. S1 to S4] shows that the bands that drive the p-type transport are of Yb and Sb character with minimal contribution from Zn^2+^. Crystal orbital Hamilton population (COHP) analysis of the Yb_14_*M*Sb_11_ (*M* = Al, Mn, Mg, and Zn) series (fig. S2) shows that in all cases, the top of the valence band is dominated by Yb-Sb and Sb-Sb interactions with little to no contribution from *M*-Sb states. With this realization, it is of little surprise that the band structures share so many similarities at the valence band edge, and one would expect similar transport properties in these systems independent of the 2+ metal (Mn, Mg, and Zn).

### Phase stability and synthesis of Yb_14_ZnSb_11_

Despite the similarities to Yb_14_MgSb_11_ and Yb_14_MnSb_11_, Yb_14_ZnSb_11_ has been reported thus far to exhibit poor thermoelectric performance with low electrical resistivity, low Seebeck coefficients, and high thermal conductivity (*7*). This is in clear contradiction with our first-principles band structure analysis. More recently, high efficiencies have been reported for the Yb_14−*x*_La*_x_*ZnSb_11_ and Yb_14−*x*_Y*_x_*ZnSb_11_ phases (*13*). These conflicting results suggest that there is something amiss in past work on Yb_14_ZnSb_11_ (*7*). Systematic studies of Yb_14_MnSb_11_, the first compound of this structure type to show exceptional thermoelectric properties (*14*), have shown that the properties can be affected by impurities (*2*). In the original report on Yb_14_ZnSb_11_ as part of the solid solution Yb_14_Mn_1−*x*_ZnSb_11_, the phase purity of the Yb_14_ZnSb_11_ pressed pellet sample was not characterized (*7*). In this report, the authors state that there is increasing amounts of Yb_9_Zn_4+*x*_Sb_4_ with increasing *x* in the solid solution Yb_14_Mn_1−*x*_Zn*_x_*Sb_11_. The transport and thermal stability properties reported for Yb_14_ZnSb_11_ match quite well with that reported for polycrystalline Yb_9_Zn_4+*x*_Sb_9_ (*15*). This suggests that the low Seebeck coefficient and relatively high thermal conductivities previously reported for Yb_14_ZnSb_11_ are due to a sizeable Yb_9_Zn_4+*x*_Sb_9_ impurity in the sample. The effective removal of impurities in other structure types has been shown to lead to higher figures of merit for thermoelectric materials (*1*–*3*, *16*, *17*), and we show here that this is also true in the case of Yb_14_ZnSb_11_.

**Fig. 3.**
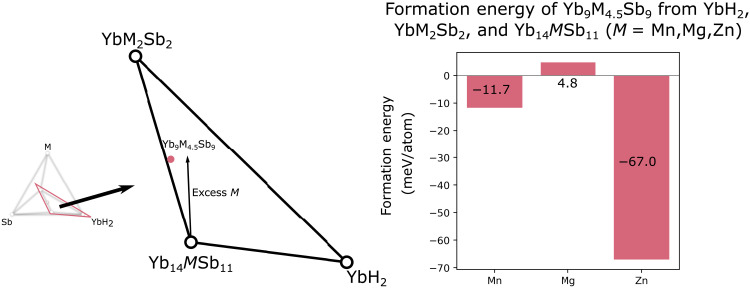
Relevant ternary phases in the Yb-*M*-Sb composition space. A diagram shows the positions of relevant ternary phases in the Yb-*M*-Sb composition space and the computed formation energy of Yb_9_*M*_4.5_Sb_9_, computed from DFT at *T* = 0 K and μ_H_ = −4 eV. From the formation energies, the formation of Yb_9_Zn_4.5_Sb_9_ is strongly energetically favored. Thus, in a synthetic route that uses a large excess of *M* (moving the overall composition closer to Yb_9_M_4.5_Sb_9_), an impurity phase could form with *M* = Zn but is less favored in the cases where *M* = Mn or Mg.

In early work on Yb_14_ZnSb_11_, Sn flux was used to grow single crystals from a combination of the elements (*7*, *18*, *19*). In these reports, reactions were performed with a large excess of Zn, in the ratios 14:6:11:86 (Yb:Zn:Sb:Sn). While this approach was very successful in growing single crystals (*18*, *19*), the excess of Zn moves the composition of the reaction closer to an adjacent ternary-phase Yb_9_Zn_4+*x*_Sb_9_ (*15*, *20*) (see Fig. 3, black arrow from Yb_14_*M*Sb_11_). As a result, Yb_9_Zn_4+*x*_Sb_9_ becomes a competing phase and a possible impurity when considering a large-scale flux growth of crystals (*7*). When considering the synthesis of polycrystalline Yb_14_ZnSb_11_, Zn dispersity becomes an even greater issue. As opposed to a flux growth in which atoms are highly mobile in a liquidous melt, here, the high melting points of the compounds that occupy this region of phase space prevent the formation of a melt, and atomic movement is reliant on solid-state diffusion. The diffusion process is slow, which makes the dispersity of elements within the reaction mixture even more important. In the Mg- and Mn-containing analogs of Yb_14_*M*Sb_11_, polycrystalline samples were prepared from the elements with an excess of *M* that ranged from 5% for Mn to 20% excess in the Mg case (*1*, *2*). The excess of *M* resulted in the removal of Yb_11_Sb_10_ impurities and an improvement in thermoelectric properties (*1*, *2*, *21*). Initial work on polycrystalline Yb_14−*x*_RE*_x_*ZnSb_11_ (RE = La, Y) took a similar approach, employing 100% excess of Zn (*13*). It is unexpected and notable that no Yb_9_Mn_4+*x*_Sb_9_ and Yb_9_Mg_4+*x*_Sb_9_ have ever been observed to the contrary of Yb_9_Zn_4+*x*_Sb_9_. We turn to DFT to try to rationalize this observation. Figure 3 compares the reaction energy of Yb_9_*M*_4.5_Sb_9_ in YbM_2_Sb_2_, Yb_14_*M*Sb_11_ and YbH_2_, focusing on the part of the phase diagram most relevant to *M* excess in Yb_14_*M*Sb_11_. All computations compare compounds with Yb in a +2 state as we want to avoid the challenging treatment by DFT of mixed oxidation states of the rare-earth Yb. A negative reaction energy indicates a stable Yb_9_M_4.5_Sb_9_ (i.e., which does not decompose in the three end members of the triangle). Figure 3 clearly indicates that Yb_9_Zn_4.5_Sb_9_ is much more favored energetically than Yb_9_Mn_4+*x*_Sb_9_ and Yb_9_Mg_4+*x*_Sb_9_. This explains why the Mn and Mg analogs could be formed with metal excess without major impurity formation and that the Zn 14-1-11 compound is inherently more difficult to form because of the competing Yb_9_*M*_4+*x*_Sb_9_ phase.

**Fig. 4.**
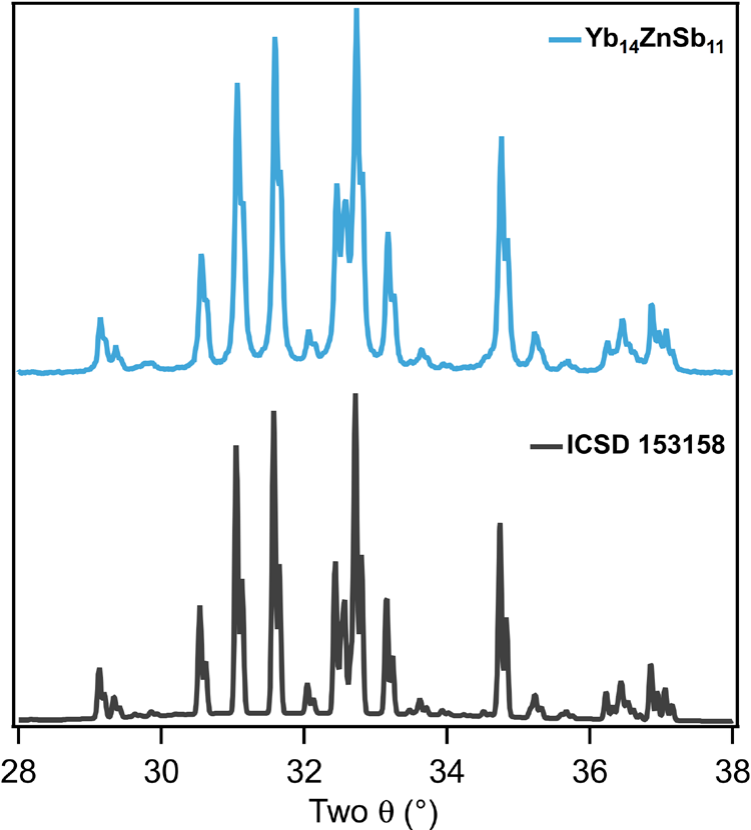
Experimental and simulated powder diffraction data for Yb_14_ZnSb_11_. The PXRD pattern for Yb_14_ZnSb_11_ (blue) synthesized from the stoichiometric reaction of the binaries, YbH_2_, Yb_4_Sb_3_, and ZnSb, and the calculated diffraction pattern from the single-crystal structure (black) (*18*). Full Rietveld refinement is provided in figs. S7 to S9 and lattice parameters in table S1.

The challenge with Yb_14_ZnSb_11_ is therefore to design a synthetic route that homogenizes the reactants while staying close to stoichiometry to avoid forming Yb_9_*M*_4+*x*_Sb_9_. Inspired by previous work on the synthesis of Yb_14_MnSb_11_ and Yb_14_MgSb_11_ (*3*), we used the binary phases, YbH_2_, Yb_4_Sb_3_, and ZnSb intimately mixed on stoichiometry to form 14-1-11, followed by reaction and consolidation in a two-step Spark Plasma Sintering (SPS) process. This route maintains stoichiometry and improves reaction interfaces required for direct formation of the targeted ternary phase instead of binary intermediates. The YbH_2_ not only provides a high-dispersity source of Yb to provide a stoichiometric reaction but also provides a partially reducing atmosphere as the hydride decomposes to elemental Yb and H_2_ gas at elevated temperatures (*1*). A slice of the densified pellet used in all the thermoelectric measurements described below was analyzed by room temperature powder x-ray diffraction (PXRD) and is shown in Fig. 4. All reflections were indexed as the tetragonal *I*4_1_*/acd* phase Yb_14_ZnSb_11_ without any unassigned intensities that could be attributed to Yb_2_O_3_, Yb_11_Sb_10_, or Yb_9_Zn_4+*x*_Sb_9_.

The unit cell parameters determined from Rietveld refinement of phase-pure Yb_14_ZnSb_11_ were *a =* 16.6105(1) Å and *c* = 21.9554(2) Å, with a unit cell volume of 6057.65(6) Å^3^. This is larger than what was previously reported for single-crystal samples [*a =* 16.562(3) Å, *c* = 21.859(5) Å, *V* = 5995.9(16) Å^3^ collected at 90 K] (*18*, *19*) and likely be due to data collection temperature. Elemental analysis by energy-dispersive spectroscopy (EDS) confirmed that the sample was phase-pure Yb_14_ZnSb_11_ (fig. S10). The lattice parameters of multiple samples of polycrystalline Yb_14_ZnSb_11_ prepared by this route described above and from the elements are in good agreement. Using the same method using different batches of Yb_4_Sb_3_ showed phase-pure Yb_14_ZnSb_11_ and confirms that the reproducibility of this route is high (see figs. S7 to S9 and table S1).

### Thermal stability and conductivity

Previous literature on Yb_14_ZnSb_11_ (*7*) suggested a possible decomposition of the phase above 800 K with a 2% mass loss and endotherm observed above that temperature. Thermometric gravimetry and differential scanning calorimetry (TG/DSC) measurements presented in this study on phase-pure Yb_14_ZnSb_11_ show thermal stability up to 1373 K, consistent with other phases of this structure type (see figs. S11 to S13 and table S2).

**Fig. 5.**
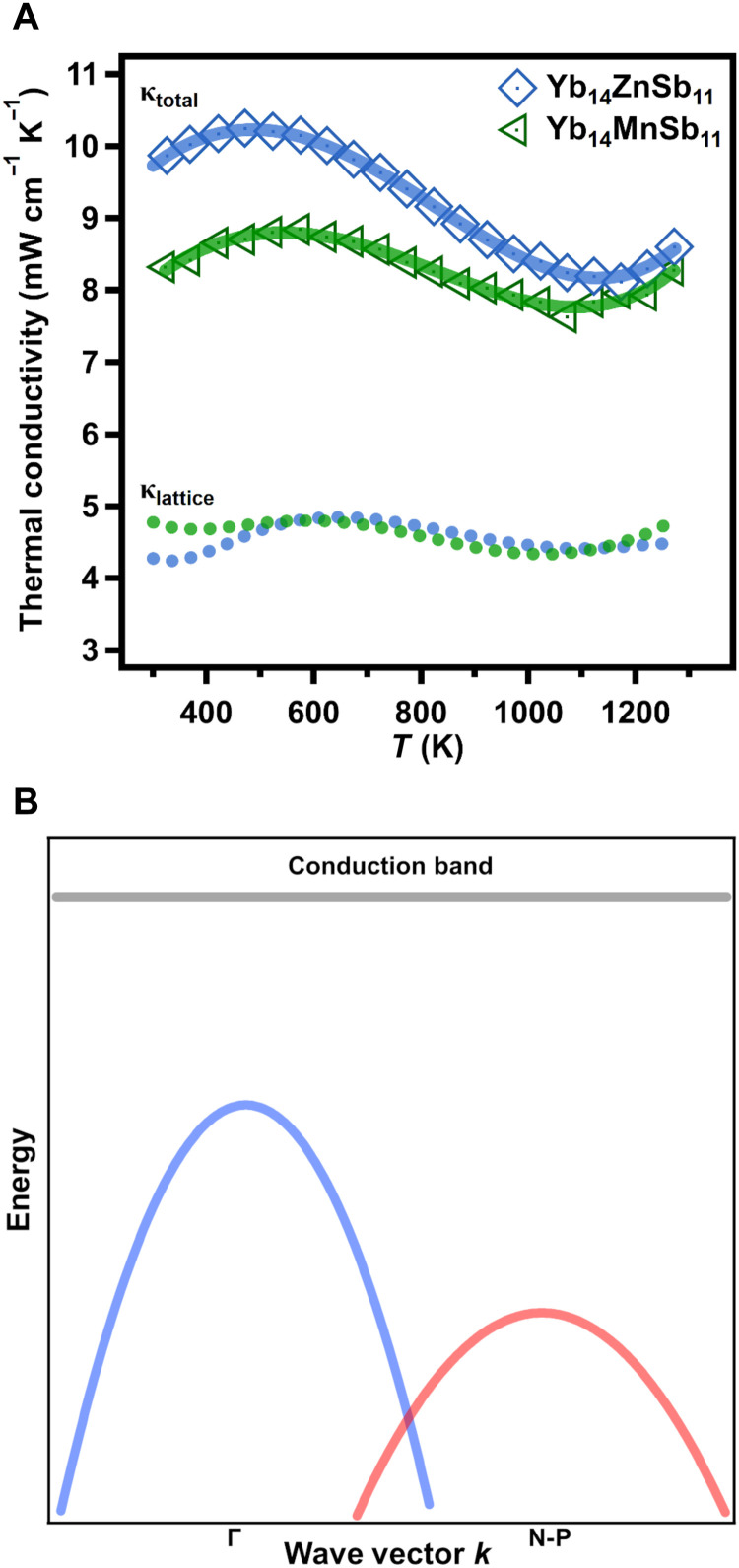
Experimental total and lattice thermal conductivity with simplified band diagram for Yb_14_ZnSb_11_. (**A**) The total thermal conductivity of Yb_14_ZnSb_11_ (blue) made from binaries compared with previously published Yb_14_MnSb_11_ made by an analogous route (*3*). The points are the average of three measurements at each temperature. The line in blue is the fit used for *zT* calculations. (**B**) A simplified model of the electronic band structure of Yb_14_*M*Sb_11_ (*M* = Mn, Zn, and Mg). Here, the light band at Γ is shown in blue, the heavy, degenerate band between N and P (N-P) is shown lower in energy in red, and the conduction band is represented as a flat gray line.

The thermal conductivity of Yb_14_ZnSb_11_ is shown in Fig. 5A, and a simplified model of the electronic band structure is shown in Fig. 5B. The thermal conductivity of Yb_14_ZnSb_11_ has the unique “S” shaped temperature dependence that is seen with other analogs of Yb_14_*M*Sb_11_ (*M* = Mn and Mg). The low temperature maximum is attributed to the distribution of carriers from the heavy to the light valence band in the two-band model previous described (*5*). At a temperature of ~1150 K, there is an increase due to the onset of bipolar conductance (*1*, *5*, *21*). In comparison to Yb_14_MnSb_11_ and Yb_14_MgSb_11_, the thermal conductivity of the Zn analog is higher over the entire temperature range. Using the Wiedemann-Franz law (κ_total_ = κ*_l_* + *L*σ*T*), the lattice thermal conductivity (κ*_l_*) can be separated from the electronic contribution (*L*σ*T*). Here, sigma (σ) is the electrical conductivity, *T* is absolute temperature, and *L* is the Lorenz number, which can be estimated using the Seebeck coefficient (*22*). Removing the electronic component from the thermal conductivity of both analogs, the higher thermal conductivity of the Zn analog can be attributed to the lower electrical resistivity (shown below) as the calculated lattice thermal conductivities of these analogs are very similar. As the method used to estimate *L* relies on the Seebeck coefficient and assumes a constant effective mass, there are slight deviations in κ*_l_* at temperature ranges where the effective mass is changing because of a change in bands contributing to transport (*5*, *22*).

### Electrical resistivity and Hall measurements

**Fig. 6.**
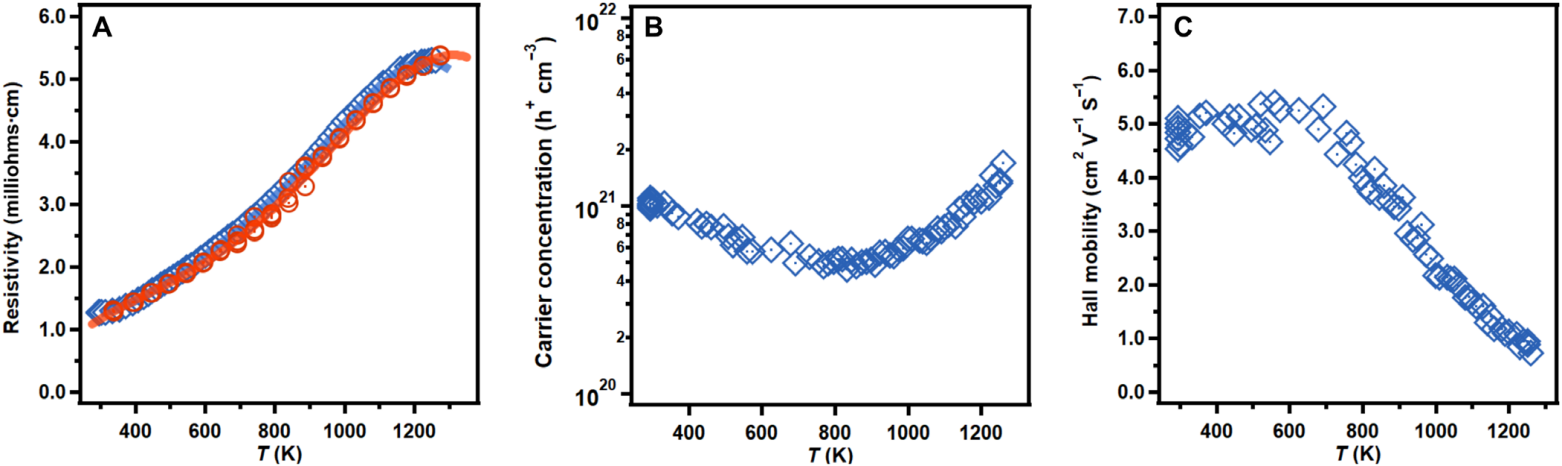
Experimental temperature-dependent transport data for Yb_14_ZnSb_11_. The (**A**) electrical resistivity of Yb_14_ZnSb_11_ from Van der Pauw (blue) and off-axis four-probe (red) measurements and Hall (**B**) carrier concentration and (**C**) mobility. The points are experimental data, and the lines are polynomial fits used for *zT* calculations.

Figure 6A provides the electrical resistivity from both Van der Pauw and off-axis four-probe measurements from room temperature to 1275 K. The measured electrical resistivity of Yb_14_ZnSb_11_ peaks to 5.30 milliohms·cm at 1250 K and then bends over because of bipolar conductance. The electrical resistivity measured by the off-axis four-probe instrumental arrangement agrees very well with the values obtained by the Van der Pauw method. The electrical resistivity here is higher than that previously reported for both single-crystal and Y- or La-substituted polycrystalline samples (*7*, *13*). The differences are attributed to impurities present in polycrystalline samples previously reported (*7*, *13*).

The Hall carrier concentration and mobility of the Yb_14_ZnSb_11_ pellet are shown in Fig. 6 (B and C). The carrier concentration starts at 1.03 × 10^21^ holes cm^−3^ at 295 K and decreases with increasing temperature to 6.47 × 10^20^ at 600 K, finally reaching a minimum of 4.88 × 10^20^ at 850 K. After that temperature, the carrier concentration begins to increase, which is attributed to the onset of bipolar conductance (*1*, *5*). The temperature dependence of the carrier concentration is consistent with the electrical resistivity measurements presented above. Yb_14_ZnSb_11_ shows comparable room temperature carrier concentrations to Yb_14_MnSb_11_ (8.06 × 10^20^ for Mn) with lower values at 600 K (*2*, *3*, *21*). The Mg analog Yb_14_MgSb_11_ exhibits lower carrier concentrations (5.3 × 10^20^ to 6.5 × 10^20^ h^+^/cm^3^ at 300 K) than its Zn counterpart across the entire temperature range (*1*, *3*).

The Hall mobility starts around 5 cm^2^ V^−1^ s^−1^ and remains relatively level until 700 K where the mobility begins to decrease rapidly down to 1 cm^2^ V^−1^ s^−1^. This temperature corresponds to the same point at which the thermal conductivity begins to decrease. This correlation suggests that the decrease in mobility is the transition of holes from the light band at Γ to the lower-mobility, heavy band between N and P (*5*). The room temperature carrier mobility of this analog is double that of Yb_14_MnSb_11_ (2.2 cm^2^ V^−1^ s^−1^), which is atttibuted to the smaller unit cell volume, leading to better orbital overlap (*2*, *14*, *21*). Despite the lower carrier concentrations, Yb_14_MgSb_11_ has slightly lower carrier mobilities (4.0 to 4.70 cm^2^ V^−1^ s^−1^) than Yb_14_ZnSb_11_ at room temperature likely due to the larger unit cell volume of the Mg analog (6150 Å^3^) (*1*, *3*).

### Seebeck coefficient

**Fig. 7.**
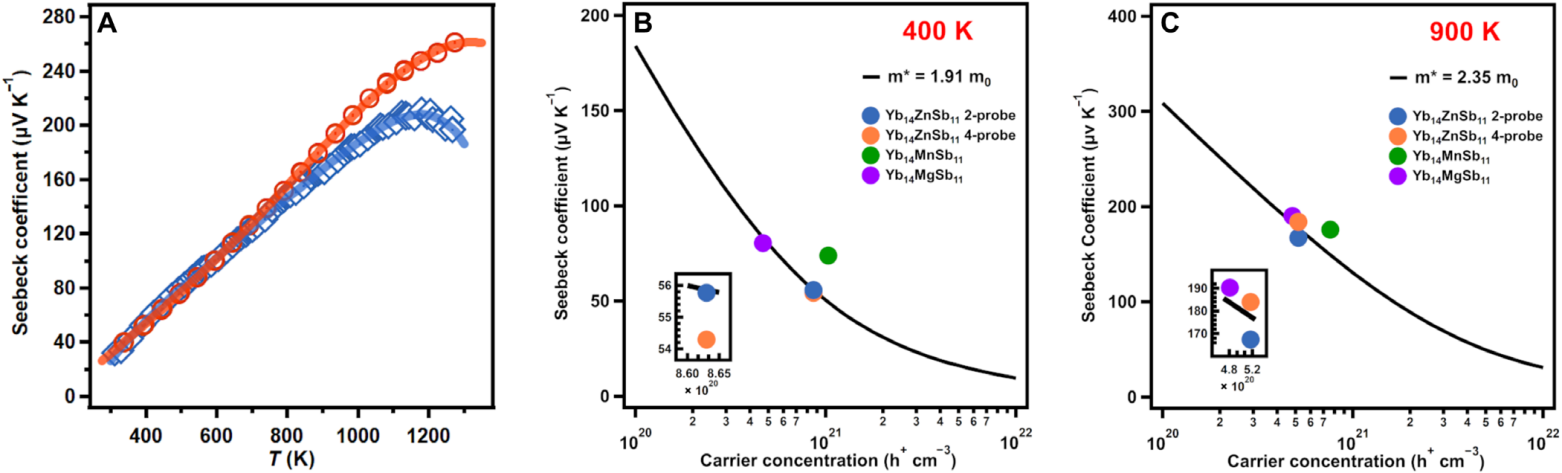
Comparison of Seebeck data obtained from two-probe and off-axis four-probe with Pisarenko plots at 400 and 900 K. (**A**) The Seebeck coefficient of Yb_14_ZnSb_11_ from two-probe (blue) and off-axis four-probe (orange) measurements. The Pisarenko plots of Yb_14_ZnSb_11_ (blue, two-probe; orange, four-probe) compared with Yb_14_MnSb_11_ (purple), and Yb_14_MgSb_11_ (green) at (**B**) 400 and (**C**) 900 K. The inset shows an expanded view to distinguish the Yb_14_ZnSb_11_ two- and four-probe data.

Figure 7A provides the temperature-dependent Seebeck coefficients of Yb_14_ZnSb_11_ from both two-probe and off-axis four-probe measurements. The two-probe Seebeck coefficient of Yb_14_ZnSb_11_ was measured on a custom instrument at JPL, allowing for direct comparison to previously reported values for Yb_14_MnSb_11_ and Yb_14_MgSb_11_ across the entire operating temperature range (*1*–*3*, *23*). The Seebeck coefficient starts at 32.27 μV K^−1^ at 311 K and increases linearly until it levels off to a peak of 211.25 μV K^−1^ at 1181 K. After that point, the Seebeck coefficients begin to decrease attributed to the onset of bipolar conductance (*22*). The temperature dependence is consistent with both the electrical resistivity and carrier concentration described above. Yb_14_ZnSb_11_ has a lower-peak Seebeck coefficient than the Mn and Mg analogs (234 and 225 μV K^−1^, respectively) (*1*, *2*, *21*). This can be attributed to the lower effective mass of the bands that make up the valence band of Yb_14_ZnSb_11_ below the Fermi level. The inflection point in the Seebeck coefficient at high temperature corresponds with an estimated bandgap of 0.50 eV by the Goldsmid-Sharp method (*24*). This agrees with the calculated bandgap of 0.43 eV and is evidence of Yb_14_ZnSb_11_ having a smaller bandgap than that of the Mg (0.53 eV) or Mn (0.59 eV) analogs and a similar gap to that of Yb_14_AlSb_11_ (0.46 eV) (*5*, *14*, *25*, *26*).

The off-axis four-probe Seebeck coefficient was measured with a commercial instrument, allowing for comparison to other materials measured by this more common method. As outlined in previous work, the measured Seebeck coefficients can be highly dependent on the method used (*3*, *22*, *27*, *28*). The off-axis four-probe method is susceptible to the systematic exaggeration of the Seebeck coefficients as temperature increases. This is commonly referred to as the “cold-finger” effect and derives from the movement of heat away from the surface of the material into the thermocouple (*22*, *27*, *29*). During the measurement, the thermocouple assembly on the hot side is at an overall lower temperature than the surface of the hot side of the material. Because of this, heat can be drawn away from the surface of the material and into the thermocouple. In addition to phonons, electronic carriers from the surface of the material are also drawn into the thermocouple probes. Both these carry thermal energy and can lower the surface temperature of the material. Despite the lowering of the surface temperature, the bulk of the sample on the hot side is at a higher temperature and a greater thermal gradient. The thermocouple probe measures the surface temperature and not the temperature of the bulk sample. This can lead to the thermal gradient being underestimated, leaving an overestimation of the Seebeck coefficient (Δ*V*/Δ*T*). This effect has been outlined as having a temperature dependence that increases with temperature (*22*).

The Seebeck coefficient measured by the off-axis four-probe method starts at approximately the same value as the two-probe measurements [39.7 μV K^−1^ at 335 K (four-probe) versus 34.0 μV K^−1^ at 328 K (two-probe)], and that trend holds for the values below 600 K. Above 600 K, the Seebeck coefficients begin to deviate from the two-probe measurement, and by 1000 K, the four-probe measurement has reached a value of 210.8 μV K^−1^. As temperature increases, the deviation between the two measurement methods increases. The four-probe measurement reaches a maximum of 261.3 μV K^−1^ at 1273 K, a 27.7% larger Seebeck coefficient than what was measured by the two-probe method (204.71 μV K^−1^ at 1261 K). A graph of the deviation in Seebeck coefficients for two-probe and off-axis four-probe as a function of temperature is provided in fig. S15. Although the Seebeck coefficients measured from the four-probe method is systematically larger at high temperatures, the calculated bandgap by the Goldsmid-Sharp method from the maximum values is only 0.66 eV (*24*). Considering that DFT has a tendency to underestimate bandgaps, this is likely within error of the calculated gap of 0.43 eV (*30*).

Pisarenko plots using the two-probe measurements at 400 and 900 K (Fig. 7, B and C) provide an effective mass of 1.91 m_0_ that increases with temperature to 2.35 m_0_ because of the contribution of the second valence band at higher temperatures, confirming the multiband nature of transport within this analog of Yb_14_*M*Sb_11_ (*5*). The effective mass at 400 K is between what can be calculated from previously reported two-probe transport data for the Mn and Mg analogs (2.89 and 1.88 m_0_), but as temperature increases and the Fermi-Dirac distribution broadens, the effective mass of the Zn analog becomes smaller than that of the other two analogs (3.45 and 2.78 m_0_) (*1*, *2*, *21*). The lower effective mass of Yb_14_ZnSb_11_ is consistent with the higher carrier mobilities seen in this analog. Using the four-probe Seebeck coefficient measurement, the same effective mass can be calculated at 400 K; however, at 900 K, the larger measured Seebeck coefficient gives a higher calculated effective mass of 2.70 m_0_. Although the higher effective mass from the four-probe is possibly inflated because of the cold-finger effect, the value is not unreasonable considering the similarities in electronic structure between the Zn, Mn, and Mg analogs.

### Thermoelectric figure of merit (*zT*)

**Fig. 8.**
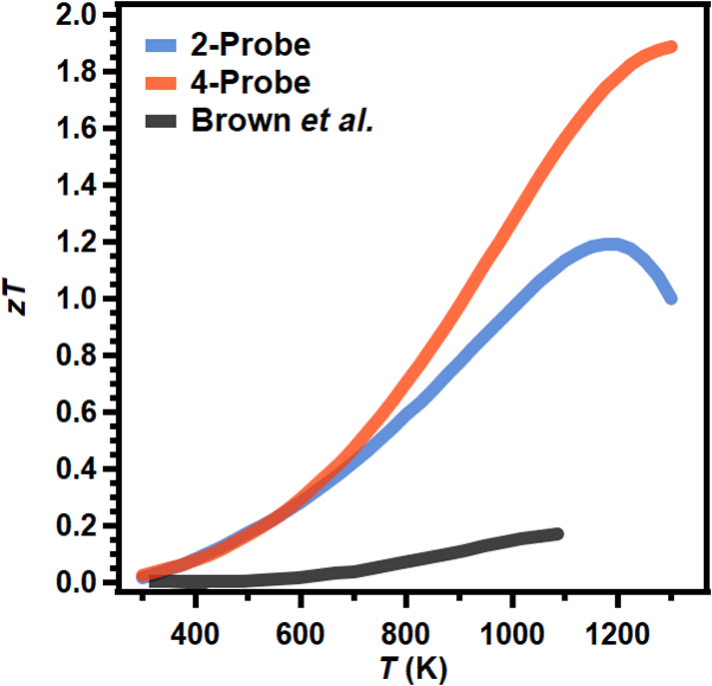
Comparison of *zT* for two-probe and off-axis four-probe Seebeck data for Yb_14_ZnSb_11_ with original published data. Unitless thermoelectric figure of merit (*zT*) of Yb_14_ZnSb_11_ from previously reported Yb_14_ZnSb_11_ (black) (*7*), compared with phase-pure pellets presented here using Seebeck coefficients measured by two-probe (blue) and four-probe (orange).

Because of the high Seebeck coefficient and low electrical resistivity, Yb_14_ZnSb_11_ shows a high power factor of 8.4 μW cm^−1^ K^−2^ from 1075 to 1150 K (fig. S16), rivaling the best reported results of Yb_14_MnSb_11_ (9.3 μW cm^−1^ K^−2^ at 1250 K) and surpassing Yb_14_MgSb_11_ (7.5 μW cm^−1^ K^−2^ from 1150 to 1200 K). Because of its complex structure, the thermal conductivity remains low to give maximum calculated *zT*s of 1.2 at 1175 K using the values from a two-probe Seebeck coefficient and Van der Pauw resistivity. This is comparable to the efficiency of Yb_14_MgSb_11_ and slightly below that of Yb_14_MnSb_11_ measured by the same methods (*1*, *2*). The maximum *zT* calculated from values measured by an off-axis four-probe reaches 1.9 at 1275 K (Fig. 8). This is the highest reported efficiency for any material of the Ca_14_AlSb_11_ structure type and rivals the highest reported *zT*s of any p-type high-temperature material. Because of the possibility of systematic errors in the measurement of high-temperature Seebeck coefficients depending on experimental setup, the *zT* calculated from the off-axis four-probe measurements is likely exaggerated but is still effective in identifying high-efficiency materials. The availability of commercial four-probe instruments makes them a powerful tool for the measurement of high-temperature Seebeck coefficients. However, the systematically large values measured by this method should be taken into consideration when making comparisons to Seebeck coefficients measured by other methods. Both experimentally determined *zT*s from this work show how the effective removal of impurities through improved synthetic methods can result in large improvements in thermoelectric performance. To confirm the reproducibility, additional samples were synthesized and measured using the two-probe Seebeck coefficient and Van der Pauw resistivity and can be found in Supplementary Materials fig. S17.

### Universality of the band structure of Ca_14_AlSb_11_ compounds

**Fig. 9.**
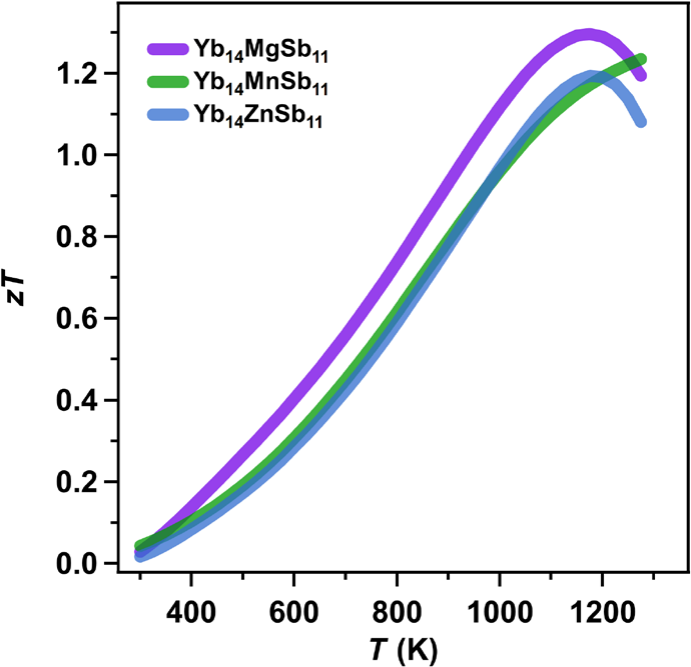
Comparison of *zTs* for Yb_14_*M*Sb_11_. The unitless thermoelectric figure of merit of Yb_14_ZnSb_11_, Yb_14_MgSb_11_, and Yb_14_MnSb_11_ made by analogous synthetic routes (*3*).

**Fig. 10.**
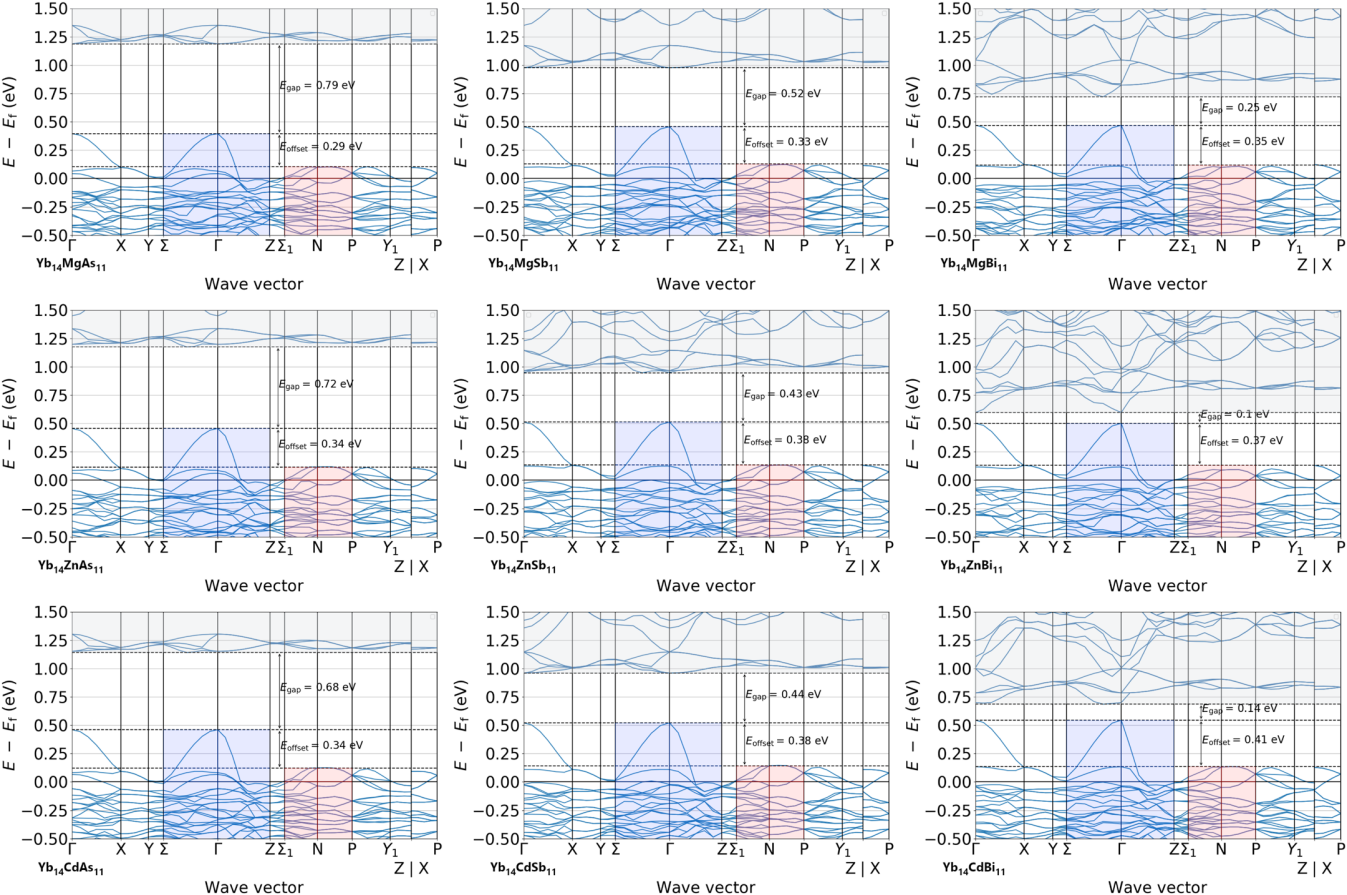
The band structures of Yb_14_*M*Pn_11_ (*M* = Mg, Zn, and Cd; Pn = As, Sb, and Bi) computed using DFT. Here, the light band at Γ is highlighted in blue, the pocket of degenerate bands between N-P is highlighted in red, and the conduction band is highlighted in gray for clarity.

Theory and experimental data have demonstrated that the Yb_14_*M*Sb_11_ with *M* = Zn, Mn, and Mg show very similar electronic structure and transport properties that result in similarly high thermoelectric performance (Fig. 9). The low *zT* observed previously (*7*) originated from impurities forming easily for the Zn analog. This raises the question of how general the excellent electronic properties for other compositions within the Ca_14_AlSb_11_ structure type are. Figure 10 shows the band structure computed within DFT for Yb_14_*M*Pn_11_ where *M* = Mg, Zn, and Cd and Pn = As, Sb, and Bi. We are not reporting on the Mn version here, which should have a more challenging electronic structure due to magnetic ordering. The similarity between the band structures of these compounds despite their widely different elemental constituents is notable. They all show the valence band features of one curvy band at Γ with a series of lower-energy flatter bands between N and P with high degeneracy that are responsible for the high thermoelectric performances. Even when Yb is replaced by Ca, the band structure retains these features (see fig. S18). This type of universality in favorable band structures is seen in other high-symmetry families of thermoelectric materials (*31*) such as lead chalcogenides (*32*, *33*), tin chalcogenides (*34*, *35*), cubic GeTe (*36*), and half Heusler phases (*37*, *38*) and in the general class of gapped metals (*39*). Further tuning and optimization of the thermoelectric performance is possible by varying the pnictogen from As to Bi, as the bandgaps and offsets between the two valence bands change in a systematic fashion. Our work indicates that many opportunities remain unexplored in the 14-1-11 field when the challenges associated with synthesis of these previously unexplored phases are overcome and that many members of the Ca_14_AlSb_11_ should exhibit exceptional thermoelectric performance if adequately doped (*5*).

## MATERIALS AND METHODS

### First-principle calculations

The electronic band structures were calculated within DFT, using the Vienna Ab initio Simulation Package (VASP) (*40*, *41*) with the Perdew-Burke-Ernzerhof (PBE)–generalized gradient approximation (GGA) functional and adopting the projector augmented-wave (PAW) (*42*, *43*) approach. The primitive structures were relaxed until the forces are less than 0.01 eV/Å. During the relaxations and static runs, the wave functions were expanded on a plane-wave basis set up to an energy cutoff of 520 eV, and the Brillouin zone was sampled using a 2 × 2 × 2 Monkhorst-Pack k-point grid. A non–self-consistent field calculation on an 8 × 8 × 8 k-point grid was performed to calculate the density of states (DOS). Then, by using the BoltzTraP (*44*, *45*) software, the eigenvalues were interpolated on a 10-times denser grid to obtain the DOS. The Yb pseudopotential has the 4f electrons frozen in the core (i.e., not as valence electrons). This was motivated by a previous x-ray photoelectron spectroscopy study, which indicated that Yb-4f states in Yb_14_MnSb_11_ and Yb_14_ZnSb_11_ are below the Fermi level by more than 0.5 and 1 eV, respectively (*19*). We also performed some tests with the PAW Yb pseudopotential providing f electrons as valence electrons and applying different Hubbard *U* values on the f states. The typical *U* values used in previous work on other Yb antimonides (*23*) lead to f states, much lower than the Fermi level, confirming that f states could be neglected. We also applied different Hubbard *U* values on the d states of Mn. Given that *U* values in range (2, 5) eV give a similar band structure (see fig. S4), we will consider in the following only the one obtained with *U* = 3 eV, which is a typical *U* value for Mn in many compounds.

Yb_14_MnSb_11_ is a ferromagnetic material with the Curie temperature (Tc) of 53 K (*46*). Transport measurements and thermoelectric operations are performed at much higher temperature leading to a paramagnetic state (*14*). In this work, all the results are performed on an antiferromagnetic spin ordering on the manganese atoms. Although the statistical effects of spin ordering are not included in this approach model, the antiferromagnetic ordering captures two important features of paramagnetism, the spin polarization and zero total magnetic moment.

We performed bonding analysis using the COHP and Lobster (*47*). The default basis functions of the “pbeVasp-Fit2015” basis set were used. The charge spilling considering all occupied states arrived at around 1.4% for all compounds. The total spilling considering both occupied and unoccupied states was around 13% for Mn and Mg compounds and 13% for Al and Zn compounds. Because the following analysis will mainly be performed for the occupied states and the charge spilling shows a very low value, this rather high total spilling should not influence our conclusions.

The Yb-*M*-Sb phase diagrams have been computed with similar parameters and with the GGA-PBE functional and with a +*U* value of 3 eV on Mn. All Mn-containing compounds were computed in both the states in which the initial magnetic moments of the Mn atoms were aligned ferromagnetic and antiferromagnetic. In all cases tested, the antiferromagnetic state had the lower final energy. Only Yb^2+^-containing compounds are considered, as modeling the different oxidation state of Yb (+2 and +3) would be challenging for DFT.This allows us to use the PAW pseudopotential with frozen f electrons as well for the phase stability computations. We did not use Yb metal as an end-member of the phase diagram but instead used YbH_2_. The Yb_9_*M*_4+*x*_Sb_9_ was calculated as Yb_9_*M*_4.5_Sb_9_, which represents one *M* interstitial atom in the Yb_9_M_4_Sb_9_ unit cell (*M* = Zn, Mn, and Mg). Pymatgen (*48*) was used for generating input files for VASP and post-processing the outputs such as plotting the band structure on the standard high-symmetry k-point path (*49*), plotting the DOS or the COHP, and building the phase diagram.

### Preparation of Yb_4_Sb_3_

Reactions were done in 10-g batches using a stoichiometric 4:3, Yb:Sb ratio. In an Ar-filled glove box [<0.5–parts per million (ppm) O_2_], Yb metal (Edgetech 99.99%) was filed using a metal rasp. These filings were added to a 55-cm^3^ tungsten carbide grinding vial (SPEX Sample Prep.) with two 12.7-mm-diameter tungsten carbide balls. Ground Sb shot (5N plus, 99.999%) was then added to the vial. The vial was closed and further sealed in mylar under Ar before being removed from the glove box. The mixture was milled for three rounds of 30 min with a thorough scrape under inert atmosphere (<0.5-ppm O_2_) in between each round using a chisel to help minimize cold welding and elemental loss as well as improve mixing. The resultant powder was then sealed under Ar in a Nb tube and then further jacketed in fused silica under vacuum (<0.5 mtorr). The reaction was annealed for 12 hours at 850°C in a tube furnace. The resultant black powder was analyzed by PXRD. In most cases, a mixture of Yb_4_Sb_3_ and Yb_11_Sb_10_ (<10% from Rietveld refinement) was observed. This mixture of phases represents a single point on the Yb-Sb phase diagram and was treated as a homogeneous distribution of those two phases. PXRD of the Yb_4_Sb_3_ used can be found in the Supplementary Materials (fig. S5).

### Preparation of ZnSb

Reactions were performed in 5-g batches using a 1:1 ratio of Zn:Sb. In an Ar-filled glove box (<0.5-ppm O_2_), Zn pieces (Columbus Chemical Industries 99.98%) and ground Sb shot were combined in a 65-cm^3^ stainless steel grinding vial with two 12.7-mm balls (SPEX Sample Prep.). The closed mill was further sealed under Ar in mylar and then milled for four rounds of an hour with 15 min off in between each round. Because Zn is much less prone to cold welding in comparison to Yb, no scrapes were used. After this milling, the majority ZnSb phase can be identified by PXRD. To remove any small amounts of unreacted Sb and Zn portions, the sample was annealed in a Nb tube under inert atmosphere at 450°C for 12 hours. The resultant gray powder was identified as phase-pure ZnSb by PXRD, which can be seen in fig. S6.

### Preparation of Yb_14_ZnSb_11_

Reactions were loaded with a total of 5 g with ZnSb, Yb_4_Sb_3_ (prepared as described above as a mixture of Yb_4_Sb_3_ and Yb_11_Sb_10_), and YbH_2_ to yield the balanced stoichiometric reaction

$$a{\mathrm{Yb}}_{4}{\mathrm{Sb}}_{3}+b{\mathrm{Yb}}_{11}{\mathrm{Sb}}_{10}+c{\text{YbH}}_{2}+\text{ZnSb}➔{\mathrm{Yb}}_{14}{\text{ZnSb}}_{11}+cH_{2}$$

Yb_11_Sb_10_ is introduced because of elemental loss in the Yb_4_Sb_3_ reaction but is easily compensated for by using the following three-variable, three-equation, system of equations to solve for coefficients *a*, *b*, and *c* (Eqs. 1 to 3). Here, the phase fractions of Yb_4_Sb_3_ and Yb_11_Sb_10_ from PXRD of that reaction mixture will be converted to mole percent (χ) using the molecular weights of the two compounds.

4a+11b+c=14 (1)

Equation 1 sets Yb content to 14

3a+10b+1=11 (2)

Equation 2 sets Sb content to 11

$$\frac{\chi_{{\mathrm{Yb}}_{4}{\mathrm{Sb}}_{3}}}{a}=\frac{\chi_{{\mathrm{Yb}}_{11}{\mathrm{Sb}}_{10}}}{b}$$ (3)

Equation 3 relates Yb_4_Sb_3_ content to Yb_11_Sb_10_ through mole fraction.

Once the above reaction was balanced, the powders were weighed in an Ar-filled glove box (<0.5-ppm O_2_) and added to a 65-cm^3^ stainless steel grinding vial with two 12.7-mm-diameter stainless steel balls. The reaction was further sealed under Ar in mylar before being removed from the glove box and milled for three rounds of 30 min. The reaction was scraped with a chisel under inert atmosphere in between rounds 2 and 3. The resultant black powder was loaded into a 12.7-mm-diameter graphite die (Cal Nano). The reaction was performed directly in an SPS instrument (Dr. Sinter Lab Jr., Fuji Corp.) by a two-stage process. The reaction was completed at an initial temperature of 600°C for 30 min under 5 kN of force and active vacuum. The progress of the reaction can be monitored with pressure as the YbH_2_ reacts, forming H_2_ gas as a by-product. After 30 min, the pressure increased to 6.5 kN, and the temperature was ramped to 850°C where it was held for another 20 min to consolidate the sample. The resultant black pellet was over 98% of the theoretical crystallographic density by the Archimedes method. This two-step process was chosen to avoid the vaporization of Zn at high temperatures before it had the opportunity to fully react.

### Powder x-ray diffraction

Phase identification and purity of polycrystalline samples was analyzed by PXRD using a Bruker D8 Eco Advanced with Cu Kα radiation (λ = 1.54 Å) and a Ni filter to remove Cu Kβ. Diffraction experiments were performed at room temperature using a zero-background off-axis quartz plate. Diffraction patterns were analyzed by Rietveld refinement using the JANA 2006 software package (*50*).

### Elemental analysis

The sample composition was analyzed by Z contrast using scanning electron microscopy (SEM, Thermo Fisher Quattro ESEM). Elemental distribution and total content were analyzed by EDS (Bruker Quantax) using a Yb_14_MgSb_11_ single crystal as the Yb and Sb standard. A Zn standard was prepared from a piece of Zn shot (99.9999%, Johnson Matthey).

SEM images of a portion of the consolidated sample after measurements show a single homogeneous phase. Backscattered electron images show no Z contrast within the sample. Any contrasted regions in the backscattered images corresponded to regions of pullout in the secondary electron images. The elemental distribution and composition of Yb_14_ZnSb_11_ were analyzed by EDS. The elemental composition from EDS was found to be Yb_14.4(5)_Zn_1.0(7)_Sb_10.5(4)_ from an average of 15 points. The minor deviations in elemental composition in comparison to nominal are due to the overlap of high-intensity Yb and Sb peaks with low-intensity Zn peaks (*51*). X-ray mapping of the sample shows a homogeneous distribution of the elements throughout the sample (fig. S10).

### Thermal stability

The thermal stability of Yb_14_ZnSb_11_ was analyzed by TG/DSC (STA 449 F3, Netzsch) using a SiC furnace and Al_2_O_3_ pans. After establishing baselines, a small piece of polished pellet was placed in the pan and heated at 10 K/min from room temperature to 1100°C and back down under Ar flow. This heating and cooling cycle was repeated four times consecutively. To avoid oxidation of the sample, four pump-purge cycles were used before starting the measurement. The purging gas flow was limited to an Ar (20 ml/min; 99.999%, Praxair) protective flow over the measurement equipment. A polished Zr ribbon was placed in a loop on top of the highest heat shield to remove minor amounts of oxygen in the purge gas flow.

### Thermal conductivity

Thermal diffusivity was measured on densified pellets using Laser Flash Analysis (Netzsch LFA 475 Microflash) under Ar flow. The fully densified pellet was sliced into a thin disk (>1.5 mm) and polished until a uniform level surface was achieved on both sides. The density of this disk was measured using the Archimedes method with toluene as the liquid. The density of the sample by the Archimedes method using toluene was 8.29(3) g/cm^3^, which is over 98% dense when considering the larger unit cell volume of this sample of Yb_14_ZnSb_11_ [*V* = 6057.65(6) Å^3^, ρ_calc_ = 8.38951(8) g/cm^3^]. Heat capacity was estimated using the molecular weight of the Yb_14_ZnSb_11_ analog relative to Yb_14_Mn_1_Sb_11_, where C_p_(Yb_14_ZnSb_11_) = C_p_(Yb_14_MnSb_11_) × MM(Yb_14_ZnSb_11_) / MM(Yb_14_MnSb_11_) (*1*, *2*, *25*). Here, MM is the molar mass of the respective compounds. The coefficient of thermal expansion for Yb_14_MnSb_11_ was used to estimate the temperature dependence of density (*2*, *25*). Thermal diffusivity as a function of temperature is provided in the Supplementary Materials (fig. S19). These were all combined to give the total thermal conductivity according to the equation: κ= *D × C*_p_ × ρ (*D* = measured diffusivity; *C*_p_ = heat capacity adjusted for molar mass; ρ = the temperature-adjusted density from Yb_14_MnSb_11_) (*52*). The uncertainty of the thermal conductivity is estimated to be ±8%, considering the uncertainties from *D*, *C*_p_, and ρ.

### Hall and resistivity measurements

Resistivity and Hall carrier concentrations were measured at the Jet Propulsion Laboratory (JPL) using the Van der Pauw method with a current of 100 mA and a 1.0-T magnet on a specialized high-temperature instrument (*53*). Pictures of the experimental setup can be seen in fig. S20. The heating and cooling curves for resistivity are provided in fig. S21. Uncertainty for Hall carrier concentrations and electrical resistivity are estimated to be ±5% (*53*).

### Two-probe Seebeck coefficient measurement

The Seebeck coefficients were measured at JPL using a custom instrument that uses the light pipe method with tungsten-niobium thermocouples under high vacuum (*23*). Pictures of the experimental setup can be seen in fig. S20. Heating and cooling curves are provided in fig. S22. Uncertainty in two-probe Seebeck coefficient is estimated at ±2% (*23*).

### Four-probe Seebeck coefficient and resistivity measurements

The Seebeck coefficient and electrical resistivity were measured on a portion of the sample after measurement at JPL. The sample was shaped into a bar (approximately 10 mm by 1 mm by 3 mm) and polished, so all sides were parallel using sandpaper. Images of the bar sample for the off-axis four-probe configuration before and after measurement along with the resistivity and Seebeck heating and cooling data are provided in figs. S22 to S24. The Seebeck coefficient and electrical resistivity were measured with an off-axis four-probe arrangement with Pt thermocouples interleaved with carbon film on the bar using a Linseis LSR-3 instrument. The measurement was performed under static He atmosphere after three prior pump/purge cycles (*P*_min_ < 20 mtorr) and heated in a high-temperature infrared furnace. A polished Zr ribbon was placed inside the susceptor to act as an oxygen sponge, protecting the sample integrity. The ribbon became blackened and brittle upon completion of the measurement. The uncertainty in electrical resistivity measurement is estimated to be ±7% (*54*). Treating the two-probe Seebeck coefficient measurement as the “correct” value, uncertainty in the off-axis four-probe measurement can be estimated as +2%/−30% (*54*). The uncertainty is asymmetric because of the cold-finger effect that leads to greater Seebeck coefficient values at elevated temperatures. The uncertainty in electrical resistivity can be estimated at ±7% considering contributions from probe spacing, tip radius, and sample dimensions (*54*).
