# Supplementary material for: Unlocking the thermoelectric potential of the Ca14AlSb11 structure type
Source: Sci Adv. 2022 Sep 7;8(36):eabq3780. doi: 10.1126/sciadv.abq3780 (PMC9451163; doi:10.1126/sciadv.abq3780)
Supplement: Supplementary file 1 — Figs. S1 to S25 Tables S1 and S2 [file sciadv.abq3780_sm.pdf]

Science Advances
AAAS

Supplementary Materials for

Unlocking the thermoelectric potential of the $Ca_{14}AlSb_{11}$ structure type

Andrew P. Justl *et al.*

Corresponding author: Geoffroy Hautier, geoffroy.t.f.hautier@dartmouth.edu;
Susan M. Kauzlarich, smkauzlarich@ucdavis.edu

Sci. Adv. **8**, eabq3780 (2022)
DOI: 10.1126/sciadv.abq3780

This PDF file includes:

Figs. S1 to S25
Tables S1 and S2

Density of States and Crystal Orbital Hamiltonian Analysis

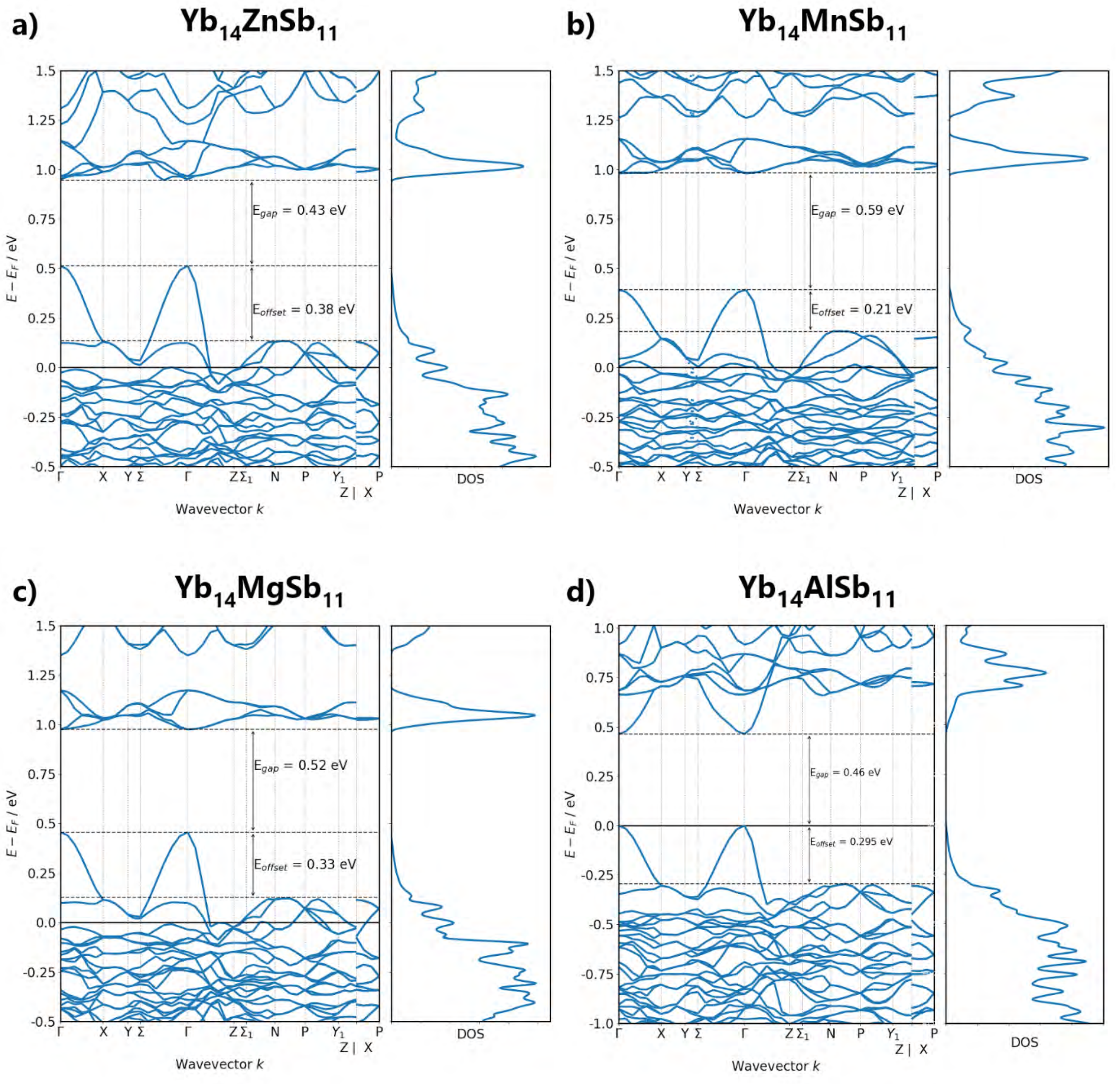

SI Figure S1. **Band structure and density of states (DOS)** a) $Yb_{14}ZnSb_{11}$, b) $Yb_{14}MnSb_{11}$, c) $Yb_{14}MgSb_{11}$, and d) $Yb_{14}AlSb_{11}$.

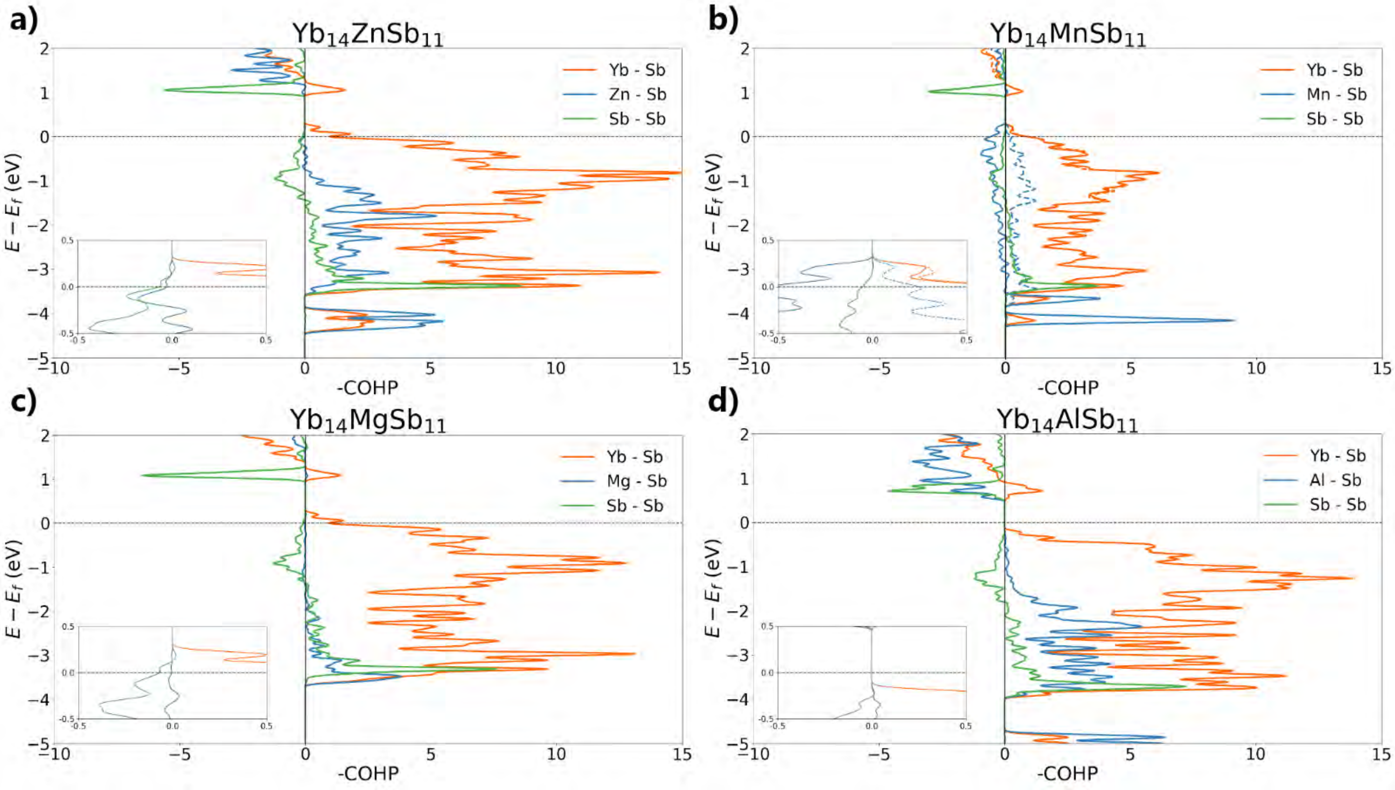

SI Figure S2. **Crystal Orbital Hamiltonian Population analysis (COHP).** a) $Yb_{14}ZnSb_{11}$, b) $Yb_{14}MnSb_{11}$, c) $Yb_{14}MgSb_{11}$, and d) $Yb_{14}AlSb_{11}$. The inset in each figure shows an expanded region near the Fermi level.

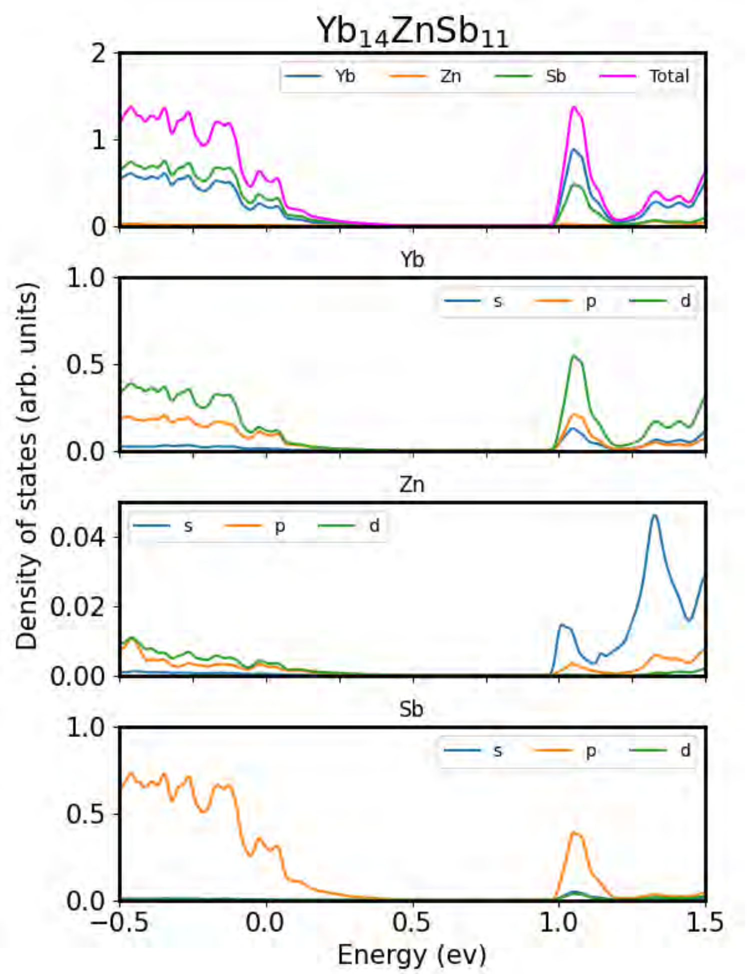

SI Figure S3. **Density of States.** The density of states (DOS) for $Yb_{14}ZnSb_{11}$ broken down by each element's contribution. The y-axis of the Zn plot has been expanded for clarity.

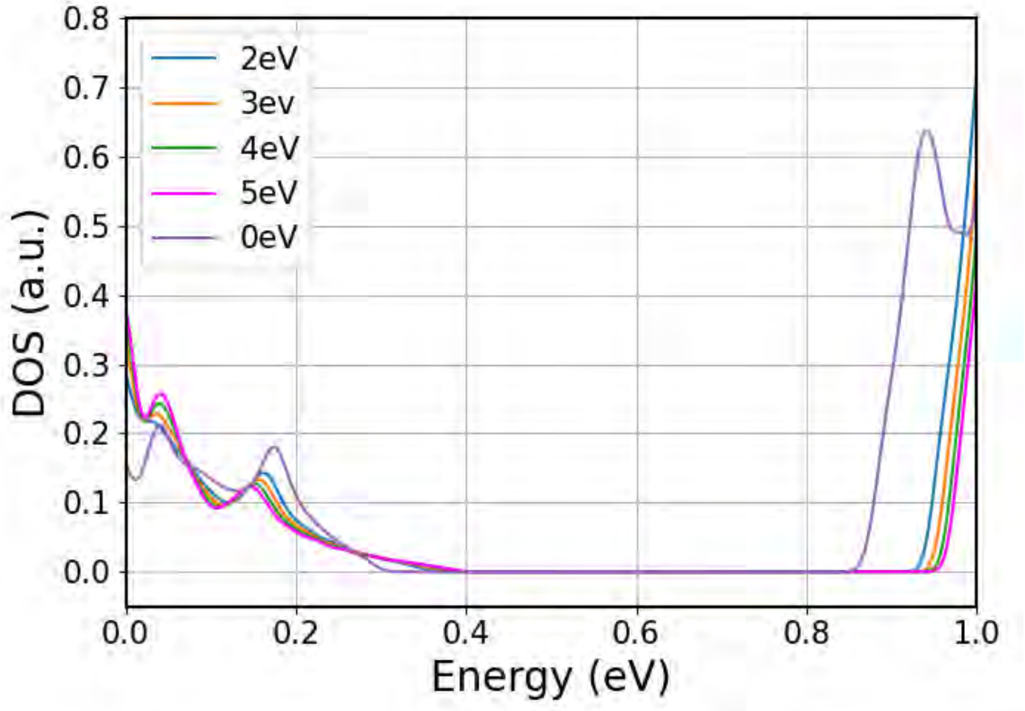

SI Figure *S4*: **Density of states for $Y_{14}MnSb_{11}$ obtained via GGA-PBE + U with different values of Hubbard U for the d orbitals of Mn.** The Fermi level is set at 0 eV.

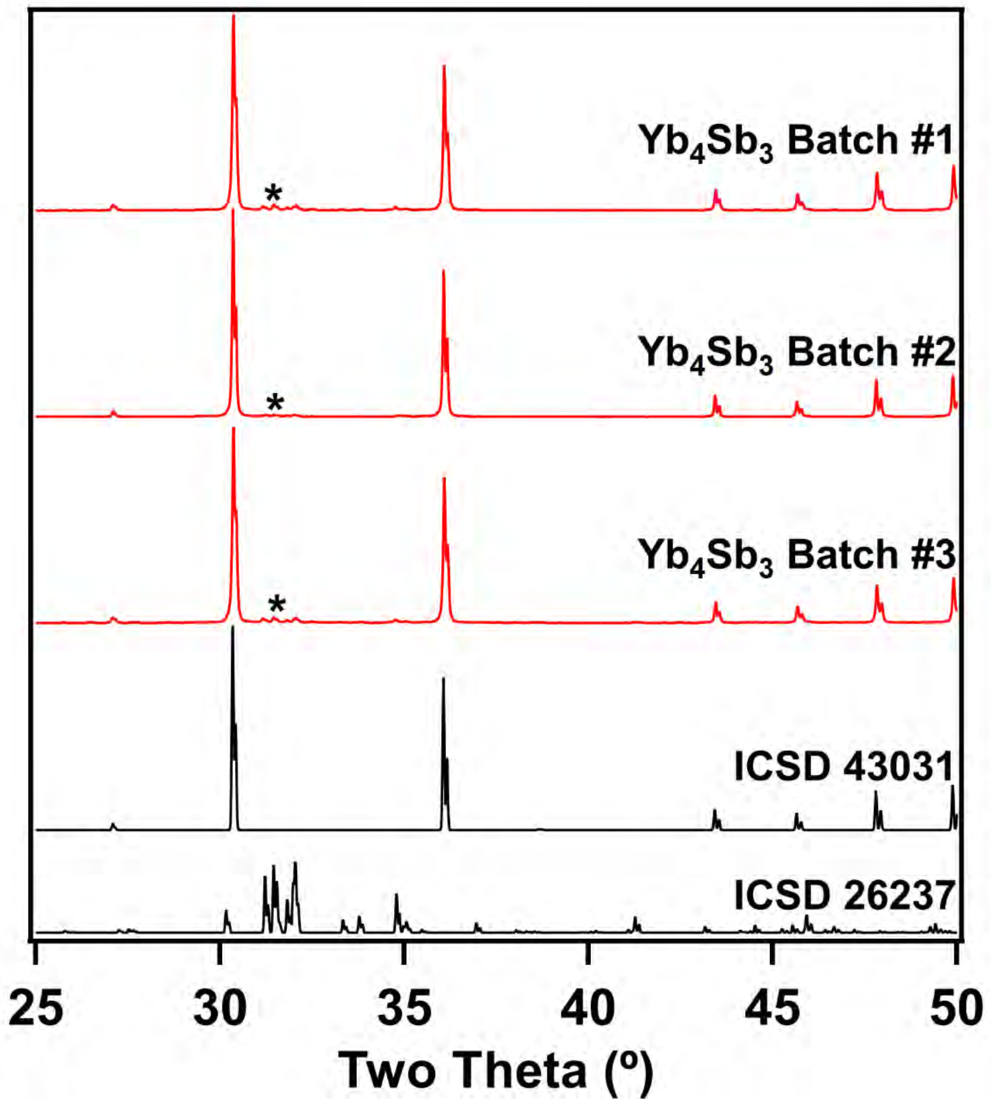

SI Figure S5. **PXRD of Yb_4Sb_3 used for reactions to synthesize $Yb_{14}ZnSb_{11}$.** PXRD of Batches of Yb_4Sb_3 (red) with reference patterns for Yb_4Sb_3 (ICSD 43031) and $Yb_{11}Sb_{10}$ (ICSD 26237) shown on the bottom in black. The minor reflections indicated with an asterisk (*) in the experimental PXRD patterns are attributed to $Yb_{11}Sb_{10}$ (9.34 (11) wt%, 7.70 (2) wt% and 4.41 (4) wt% for Batch #1, #2, and #3, respectively).

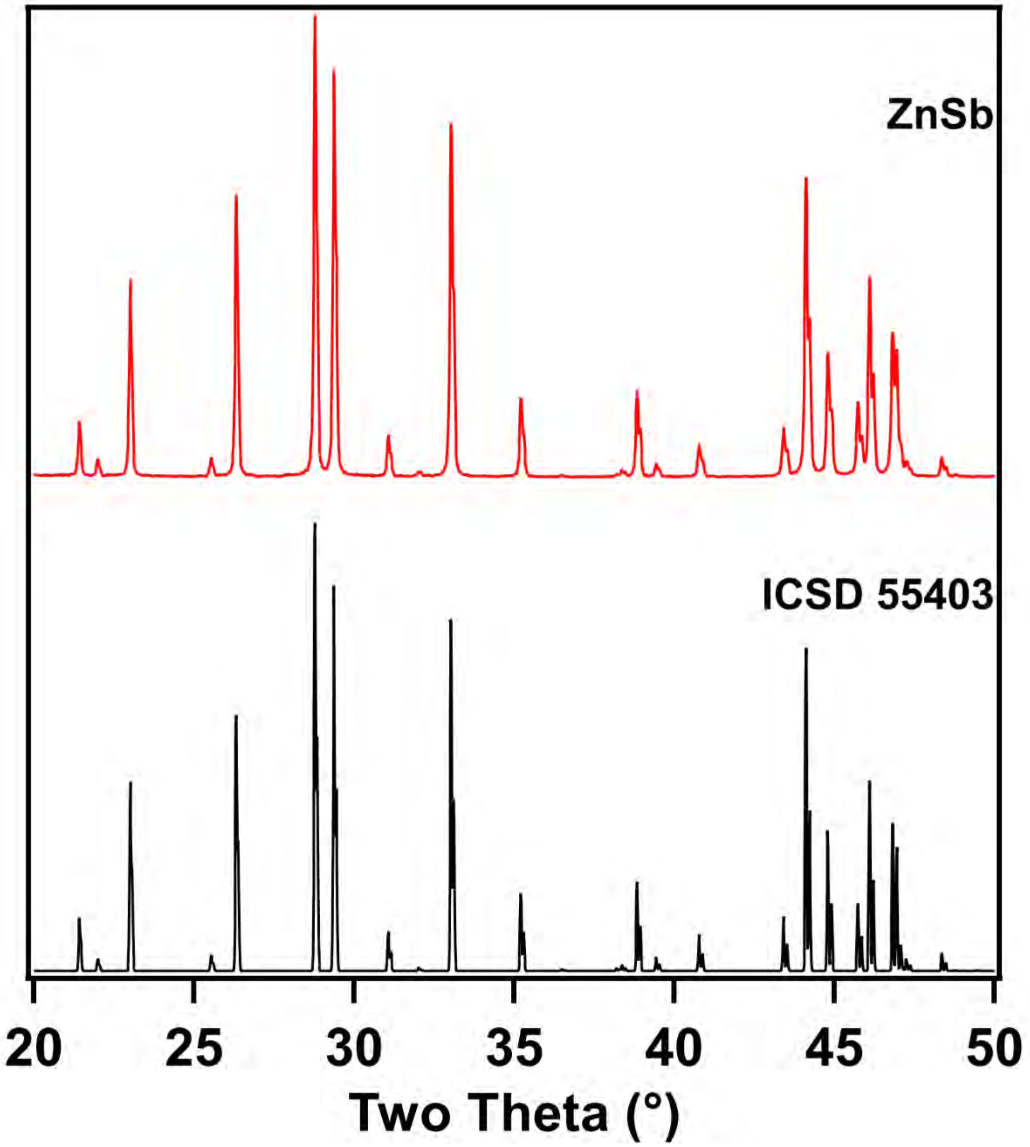

SI Figure S6. **PXRD of ZnSb.** PXRD of ZnSb (red) employed for synthesis of $Yb_{14}ZnSb_{11}$ with a reference pattern shown in black.

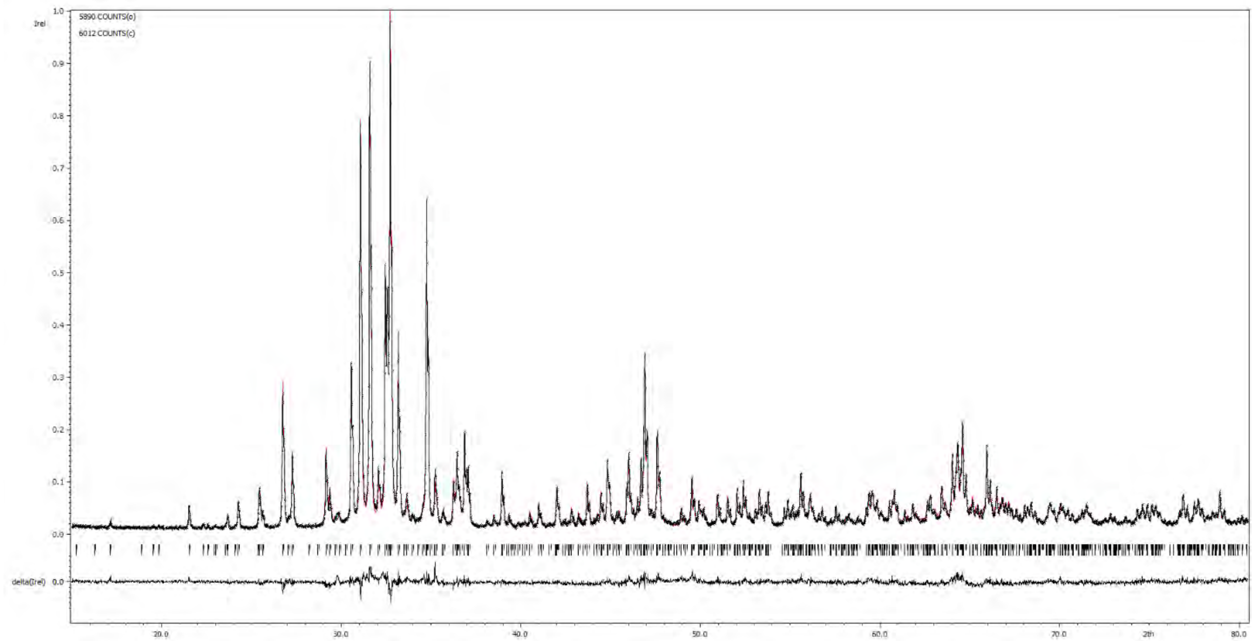

SI Figure S7. **Rietveld refinement of $Yb_{14}ZnSb_{11}$ made from YbH_2, Yb_4Sb_3, and ZnSb.** The experimental pattern is shown in black, the fit in red, and the difference is shown on the bottom in black. All intensities are well accounted for by the single $Yb_{14}ZnSb_{11}$ phase (GOF: 1.45, Rp: 6.41, wRp: 8.76).

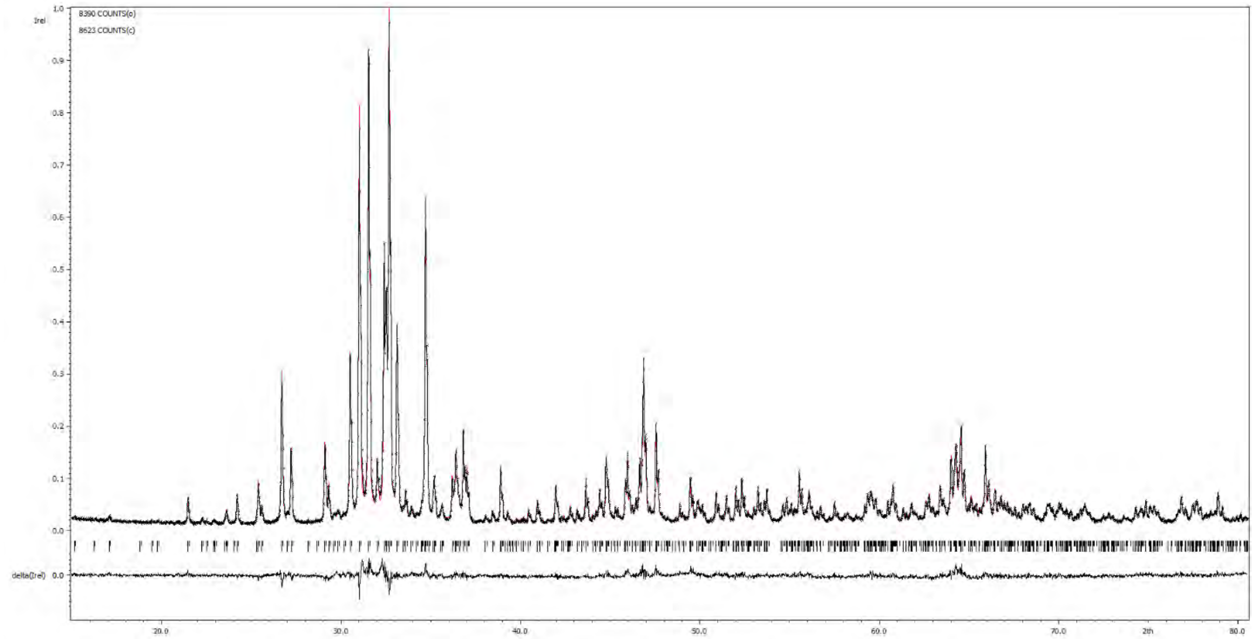

SI Figure S8. **Rietveld refinement of a second sample of $Yb_{14}ZnSb_{11}$ made from YbH_2, Yb_4Sb_3, and ZnSb.** The experimental pattern is shown in black, the fit in red, and the difference is shown on the bottom in black. All intensities are well accounted for by the single $Yb_{14}ZnSb_{11}$ phase (GOF: 1.58, Rp: 5.89, wRp: 7.96).

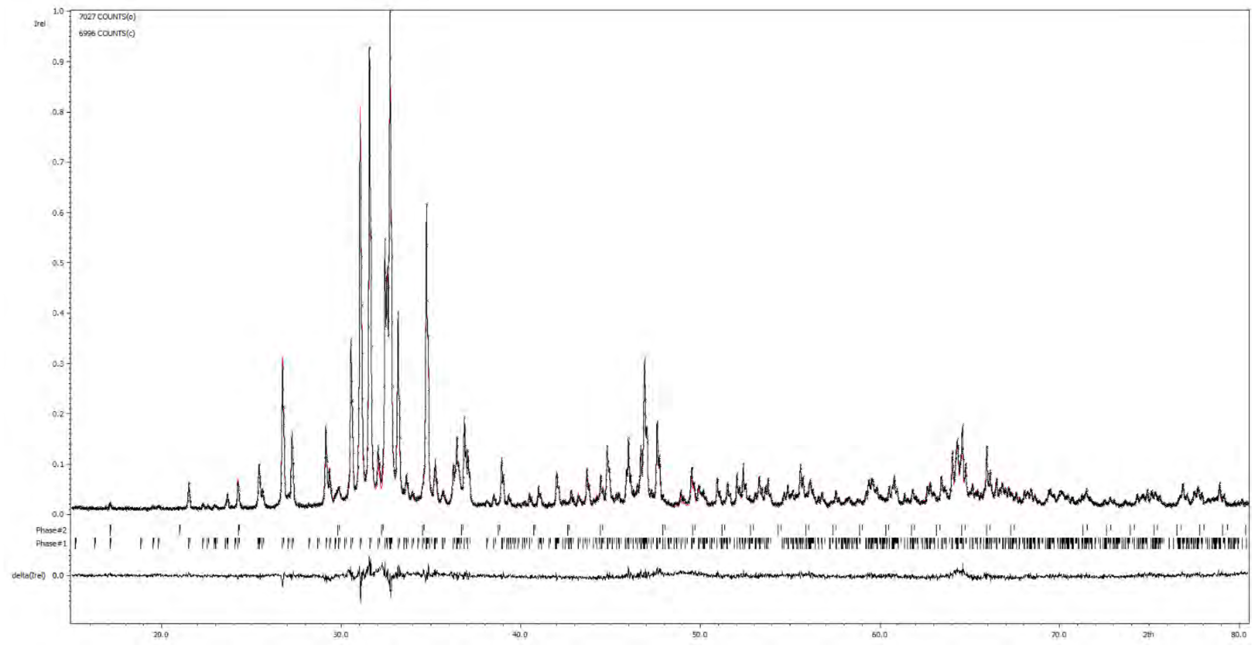

SI Figure S9. **Rietveld refinement of a third sample of $Yb_{14}ZnSb_{11}$ made from YbH_2, Yb_4Sb_3, and ZnSb.** The experimental pattern is shown in black, the fit in red, and the difference is shown on the bottom in black. All intensities are well accounted for by the single $Yb_{14}ZnSb_{11}$ phase (GOF: 1.54, Rp: 6.27, wRp: 8.62).

SI Table S1. **The Lattice Parameters from Rietveld Refinement of PXRD Patterns of Polycrystalline $Yb_{14}ZnSb_{11}$**

$Yb_{14}ZnSb_{11}$ Preparation Route	*a* (Å)	*c* (Å)	V (Å^3)
Yb_4Sb_3 - 1	16.6105(1)	21.9553(2)	6057.65(6)
Yb_4Sb_3 - 2	16.612(1)	21.9517(2)	6057.9(9)
Yb_4Sb_3 - 3	16.612(1)	21.951(2)	6058.1(8)
Elemental - 1	16.6100(9)	21.949(1)	6055.5(6)
Elemental - 2	16.6109(8)	21.9496(2)	6056.4(1)

Each sample is the result of an individual synthesis. The three reactions from Yb_4Sb_3 used different batches of Yb_4Sb_3 to help show reproducibility. Reactions from the elements used a 7% excess of Zn and showed large impurities of $Yb_{11}Sb_{10}$ and Yb_2O_3 which may minorly affect the refinement of unit cell parameters due to peak overlap. Overall, the samples made by both routes have unit cell volumes within 2 Å^3 of each other. This is within the uncertainty of the refinement.

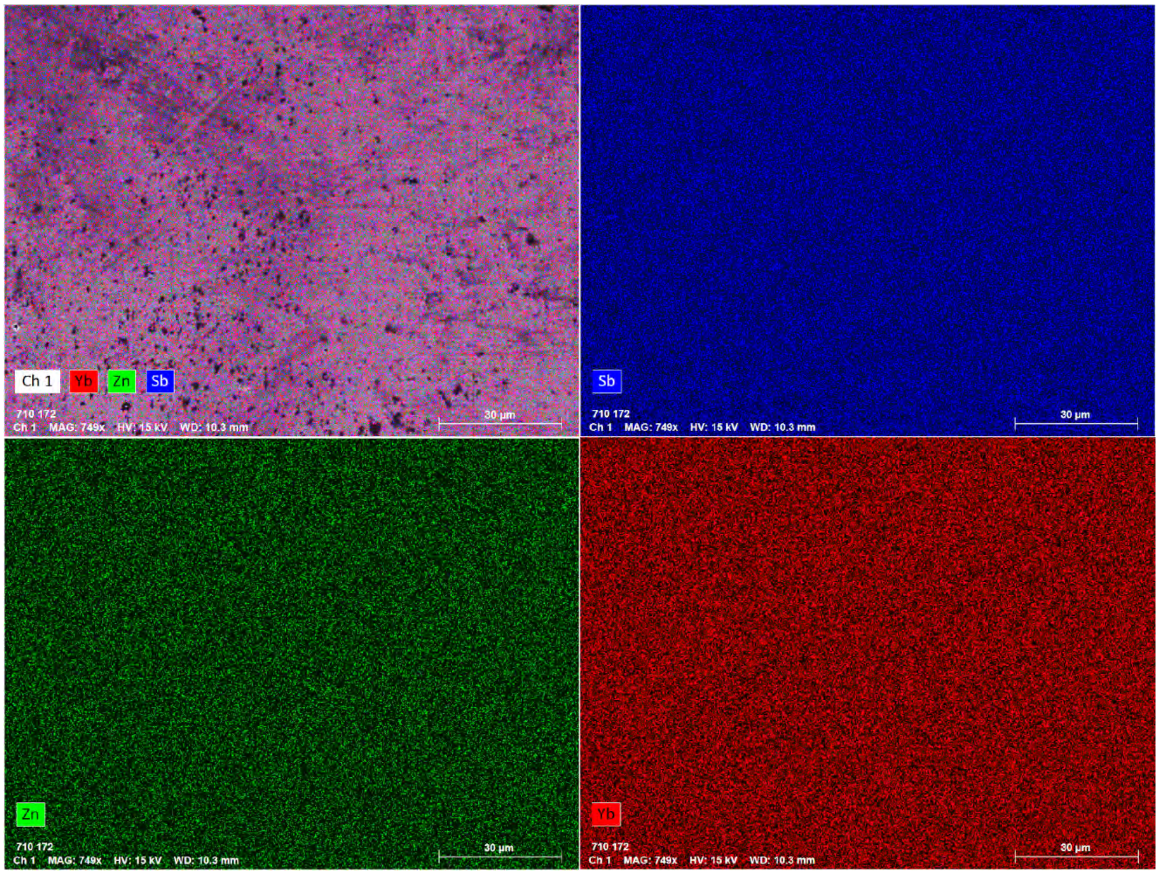

SI Figure S10. **X-ray maps of a pressed pellet of $Yb_{14}ZnSb_{11}$ after thermoelectric measurements from scanning electron microscopy energy dispersive spectroscopy.** The combined map is overlayed on a secondary electron image in the top left, Sb is in the top right (blue), Zn is in the bottom left (green), and Yb is on the bottom right (red). Although the secondary electron image shows some pullout and scratches due to poor polishing, the elemental distribution is homogenous across all elements.

SI Figure S11 shows a TG/DSC experiment of a sample of $Yb_{14}ZnSb_{11}$ cycled four times to 1375 K The TG signal shows a steady increase in mass which corresponds with the oxidation of the sample due to minor oxygen impurities in the Ar gas flow. There is a minor mass loss at 1100 K in the first cycle, but this is quickly regained and can be attributed to a small portion of the sample chipping off due to oxidation. The first cycle of the DSC measurement shows a slightly lower exotherm at low temperatures. At 700 K there is an inflection point and the exotherm increases. Repeated cycles show a larger exotherm at low temperatures and all cycles show an increase in this exotherm around 700 K. Above 1000 K, there is a slight decrease in the exotherm in the heating cycles. The feature at 700 K corresponds with the onset of high temperature oxidation of the sample as previously reported for $Yb_{14}MnSb_{11}$. Due to the use of polished Zr as a oxygen getter, the low temperature region of the first cycle would have significantly lower oxygen concentrations than future cycles. This is because the Zr surface would be passivated at low temperatures by the formation of the oxide coating. In the first cycle, the polishing of the

ribbon removed any native oxides, increasing its activity during the first cycle. As the ribbon oxidized the ability of it to react with more oxygen is lessened.

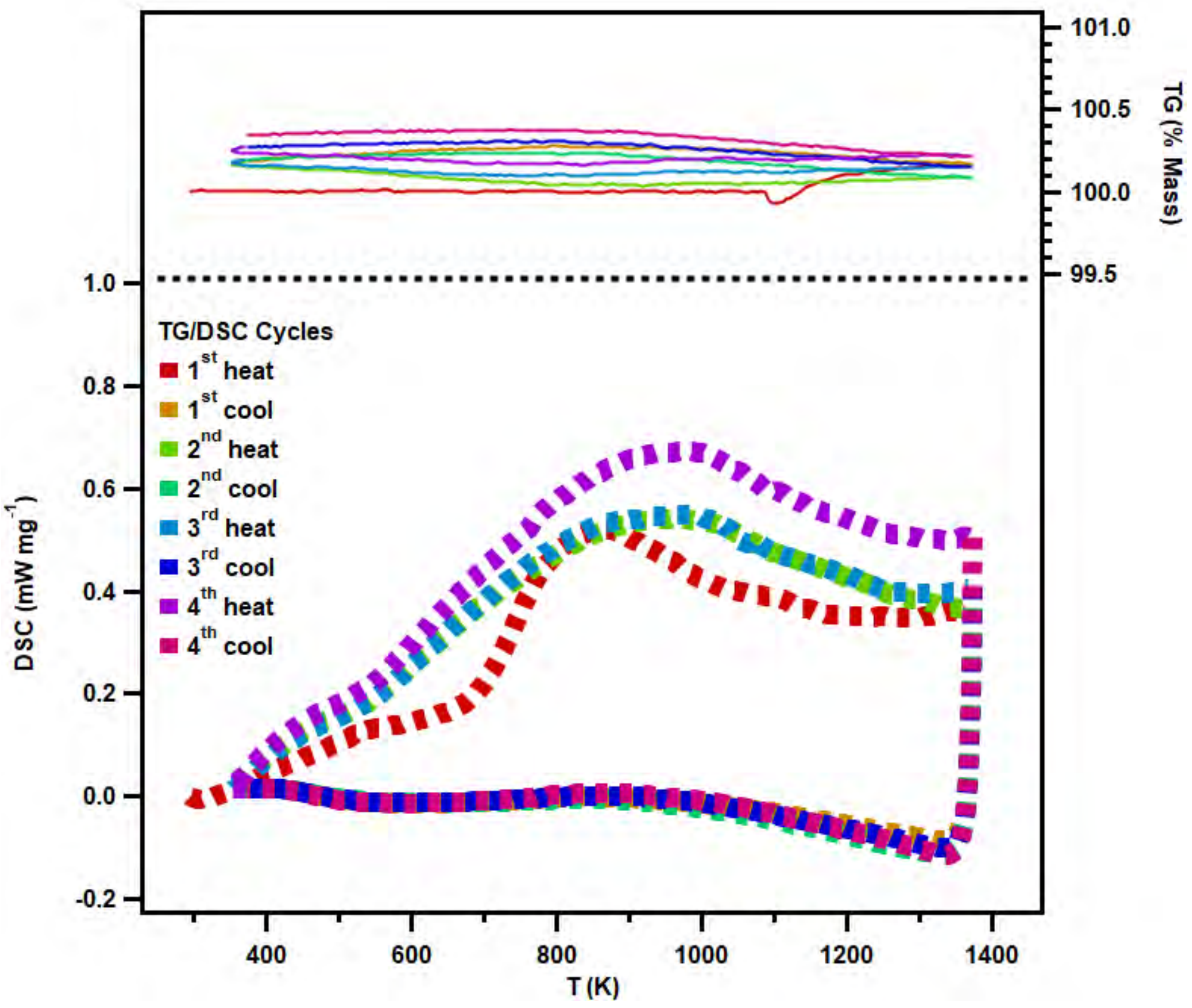

SI Figure S11. **TG/DSC of $Yb_{14}ZnSb_{11}$.** A TG/DSC experiment of a sample of $Yb_{14}ZnSb_{11}$ cycled four times to 1375 K.

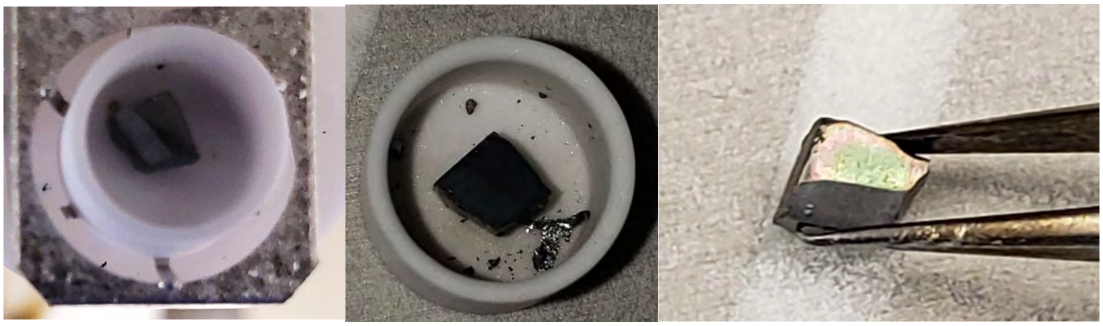

SI Figure S12. **A sample of $Yb_{14}ZnSb_{11}$ cycled to 1100°C once.** The picture on the left shows the polished sample before measuring, the middle is after measurement, and the right is the sample after measurement to show the thin film interference created by the film of oxide on the surface of the sample.

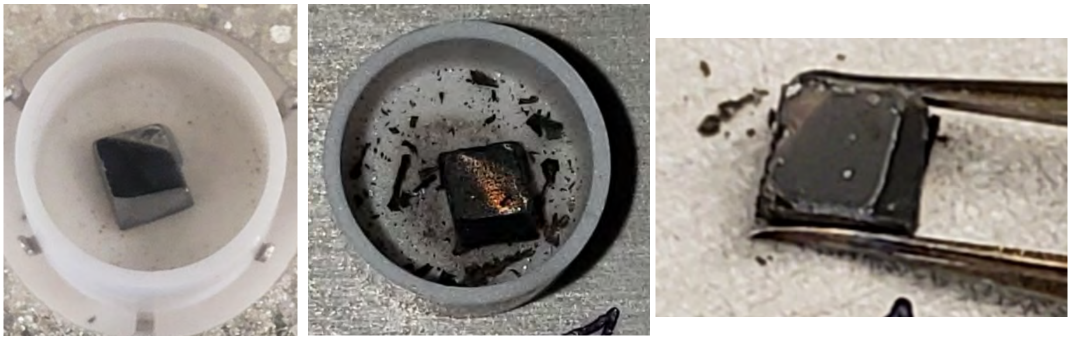

SI Figure S13. **A sample of $Yb_{14}ZnSb_{11}$ cycled from room temperature to 1100°C four times.** The left picture is the sample before measurement. The middle is post measurement and shows thin film interference of the oxide coat during flash photography. The right picture is the sample after measurement in ambient fluorescent lighting. Under less intense lighting, the thin film interference is not visible due to the thicker oxide coat.

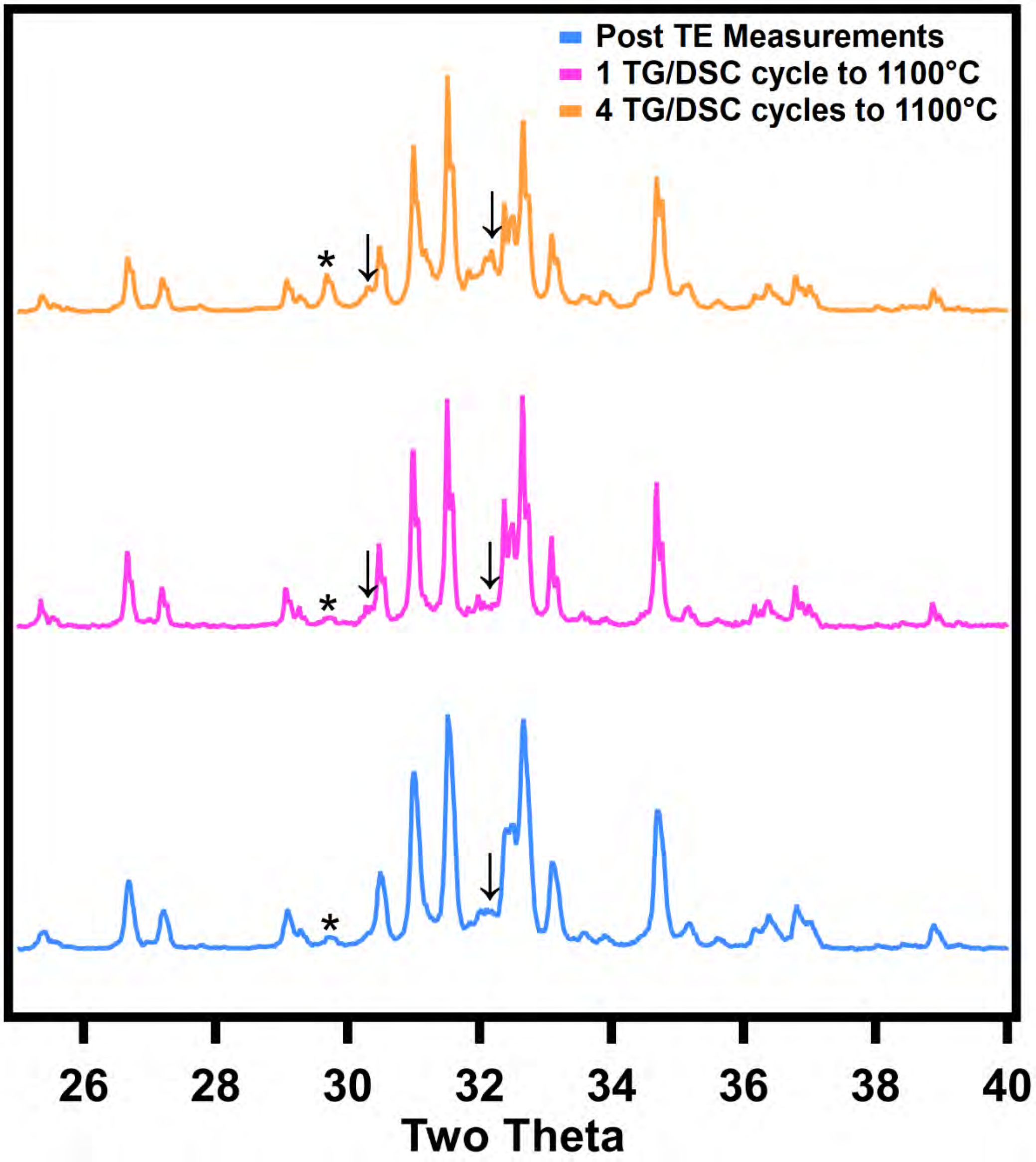

SI Figure S14. **A portion of the PXRD of $Yb_{14}ZnSb_{11}$ after heating measurements.** The powder x-ray diffraction patterns of $Yb_{14}ZnSb_{11}$ after thermoelectric measurements (blue), after a single TG/DSC cycle to 1100°C (pink), and after four TG/DSC cycles to 1100°C (orange). The two oxidation products, Yb_2O_3, and $Yb_{11}Sb_{10}$ are marked by the star and arrows respectively. The amounts of both Yb_2O_3 and $Yb_{11}Sb_{10}$ increase moving up the y axis and correspond with the high temperature oxidation of the sample. The linear relationship between Yb_2O_3 and $Yb_{11}Sb_{10}$ supports the idea that both are results of oxidation which likely occurs as $Yb_{14}ZnSb_{11} \rightarrow Yb_2O_3 + Yb_{11}Sb_{10} + ZnO$.

SI Table S2. **Rietveld Refinement Parameters from PXRD Patterns of $Yb_{14}ZnSb_{11}$ after Thermoelectric Measurements, and of Pieces Cycled in the TG/DSC from Room Temperature to 1100°C Once, and Four Times**

$Yb_{14}ZnSb_{11}$ Post Experiment	$Yb_{14}ZnSb_{11}$ (wt %)	Yb_2O_3 (wt %)	$Yb_{11}Sb_{10}$ (wt %)	GOF, Rp, wRp
TE Measurements	91.19(17) %	0.61(5) %	8.20(17) %	1.49, 7.30, 9.82
TG/DSC 1100°C x 1	89.3(2) %	0.82(8) %	9.9(2) %	1.25, 7.89, 10.37
TG/DSC 1100°C x 4	73.6(2) %	3.94(9) %	22.4(2) %	1.74, 8.33, 11.00

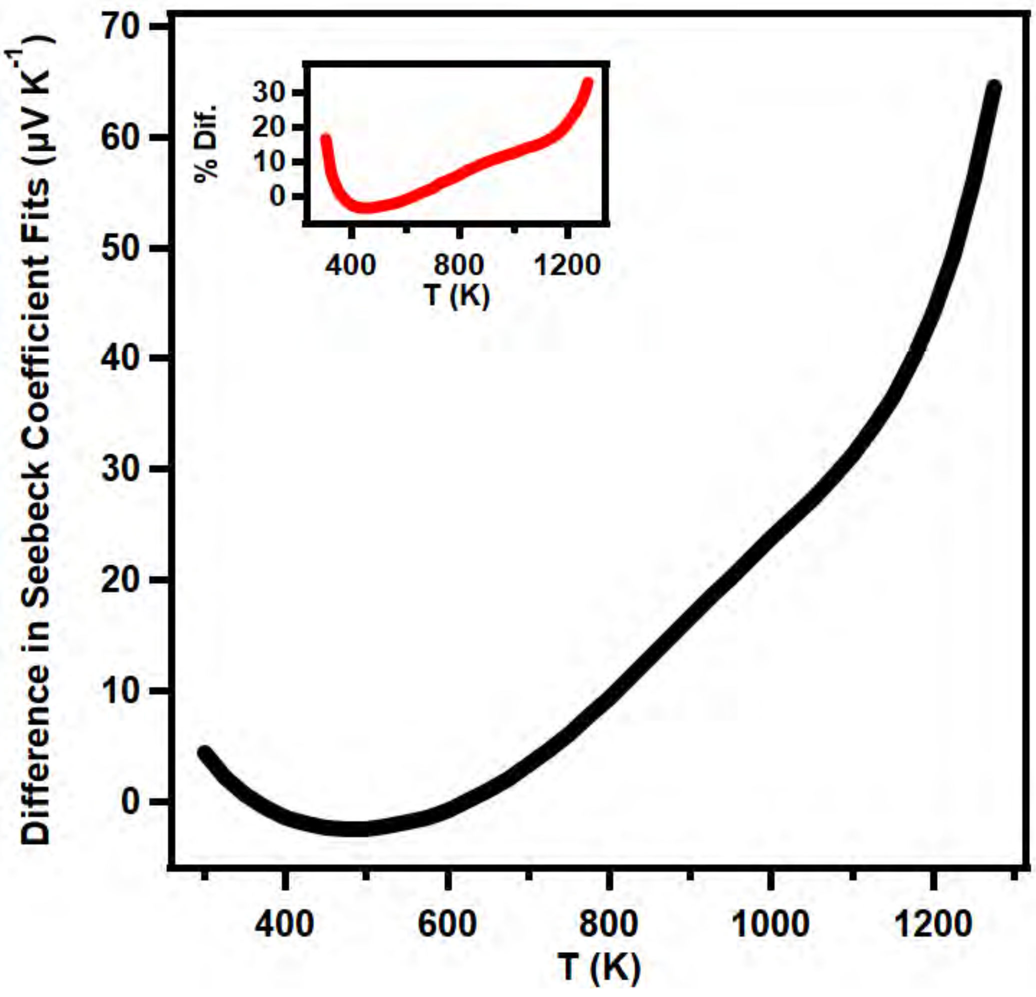

SI Figure S15. **Deviations between the fits obtained from 2-probe and 4-probe Seebeck coefficients as a function of temperature for $Yb_{14}ZnSb_{11}$ measured to 1275 K.** The black line shows absolute difference, and the red line in the inset plot shows the percent difference of the 4-probe in reference to the 2-probe measurement.

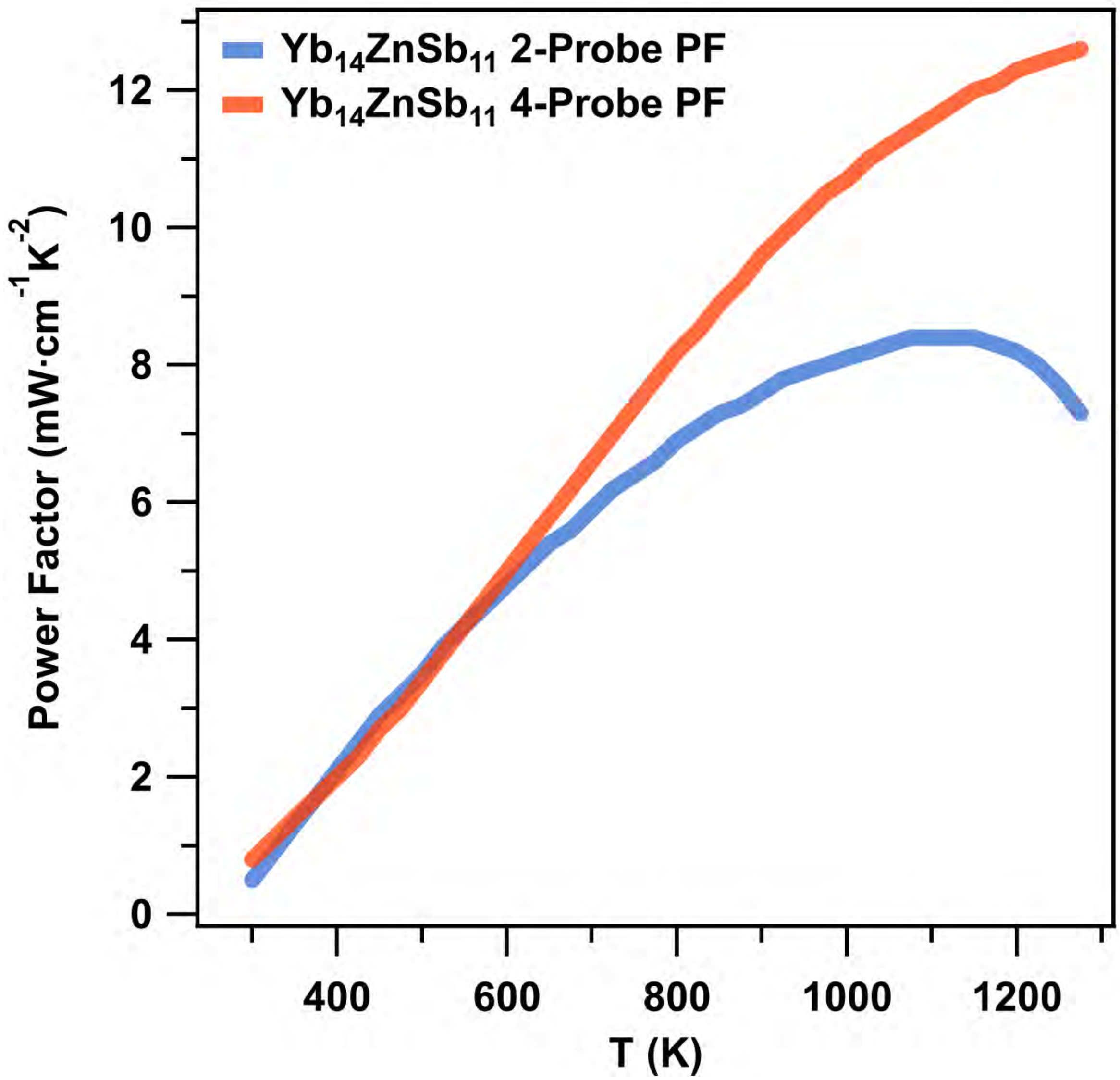

SI Figure S16. **Power factor plots.** The power factor of $Yb_{14}ZnSb_{11}$ as calculated from 2-probe (blue) and off-axis 4-probe (orange) measurements.

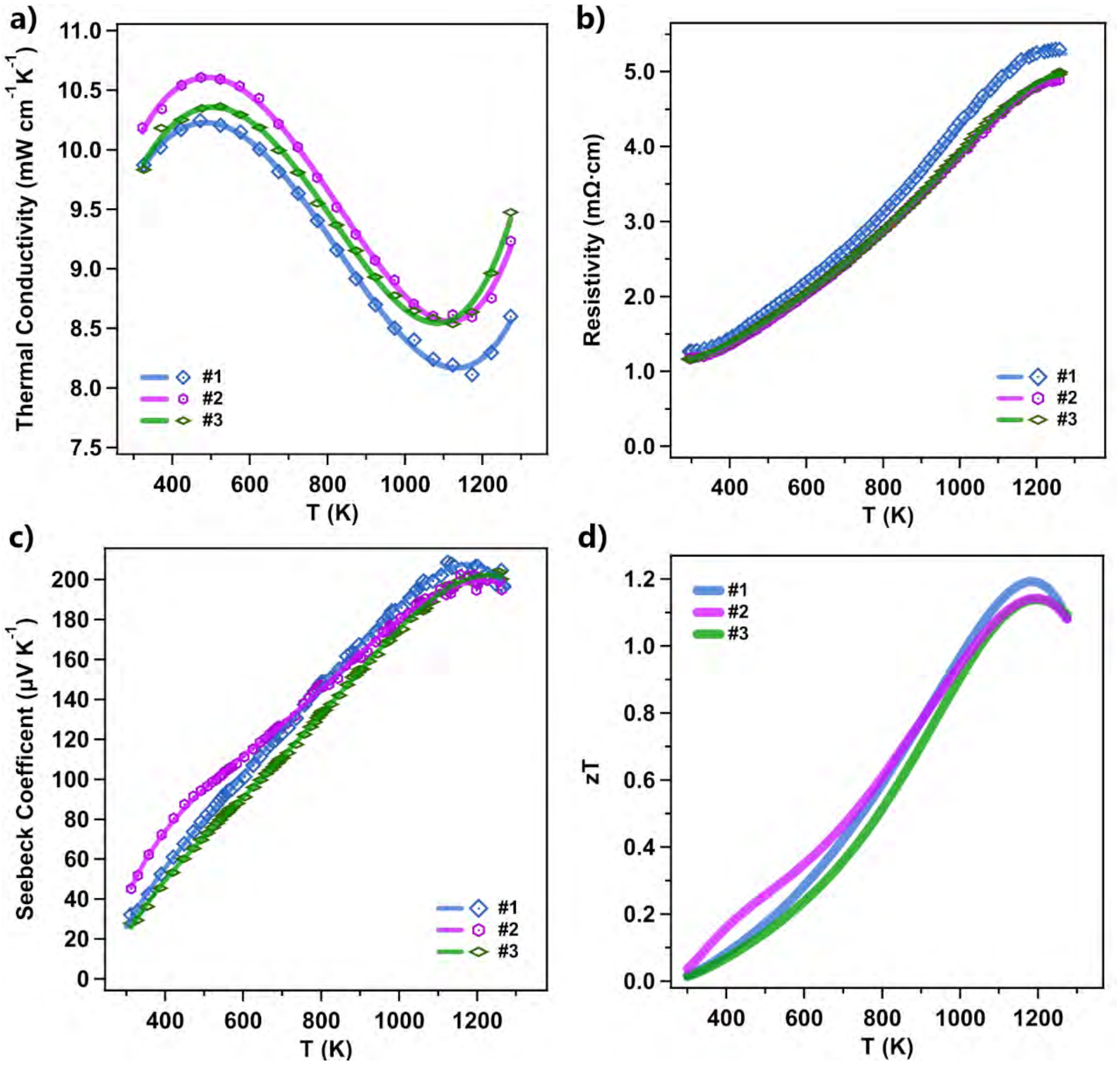

Figure S17. **Thermoelectric measurements of 3 pellets of $Yb_{14}ZnSb_{11}$.** The a) thermal conductivity, b) electrical resistivity, c) Seebeck coefficient, and d) zT of two additional pellets of $Yb_{14}ZnSb_{11}$ prepared by the same synthetic route (#1 is in the manuscript, plus 2 additional samples).

Band Structures of $Ca_{14}M Pn_{11}$

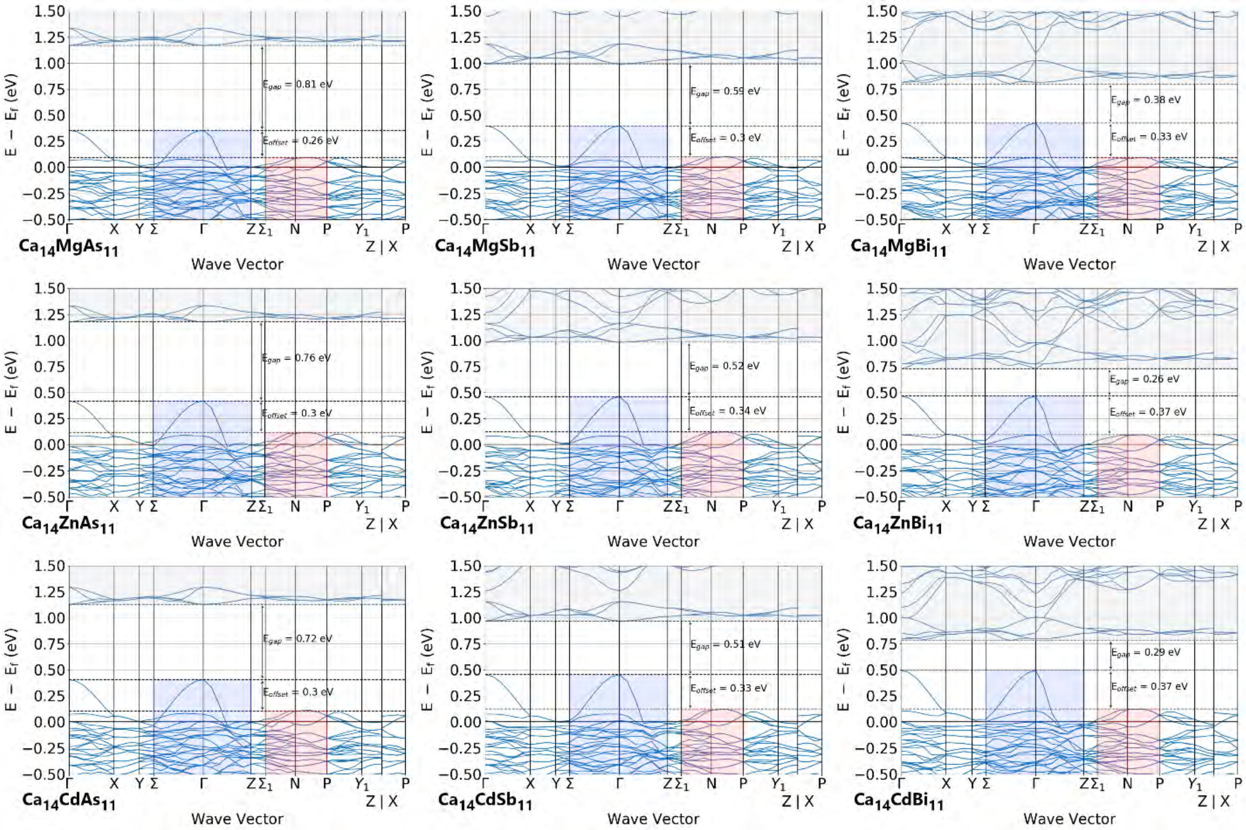

SI Figure S18. **Band structure diagrams.** The calculated band structures of $Ca_{14}M Pn_{11}$ (M = Mg, Zn, Cd; Pn = As, Sb, Bi). The first valance band can be seen highlighted in blue at Γ, the second, degenerate valance band between N and P is highlighted in red, and the valence band is highlighted in grey for clarity.

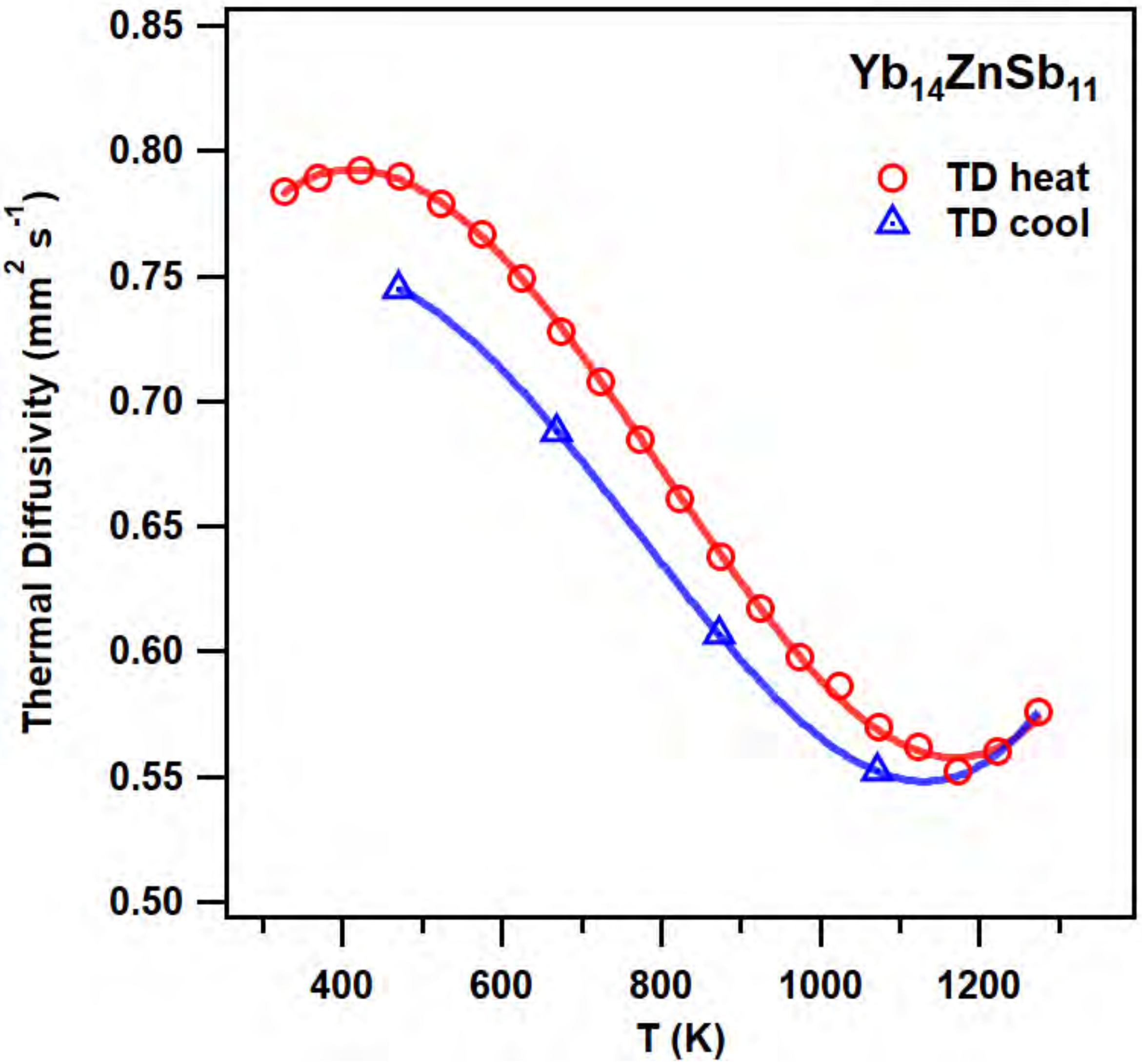

SI Figure S19. **Temperature dependent thermal diffusivity.** The thermal diffusivity of $Yb_{14}ZnSb_{11}$ on heating (red, circles) and cooling (blue, triangles). The slight decrease in thermal diffusivity on cooling can be attributed to oxidation during the high temperature portion of the measurement.

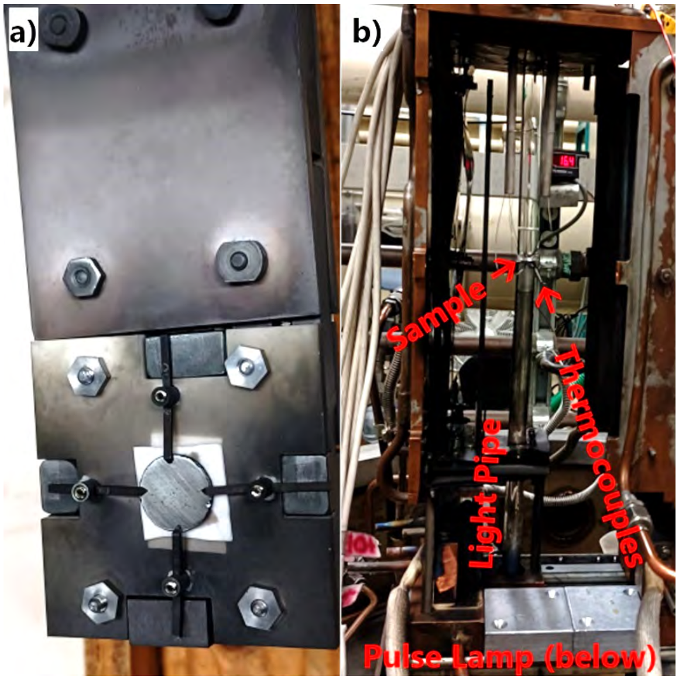

SI Figure S20. **Images of instruments at the Jet Propulsion Laboratory.** a) Van der Pauw resistivity and b) custom 2-probe Seebeck coefficient instruments. For further information on the Seebeck instrument including detailed schematics see: C. Wood, D. Zoltan, G. Stapfer, Measurement of Seebeck coefficient using a light pulse. Rev. Sci. Instrum. 56, 719–722 (1985).

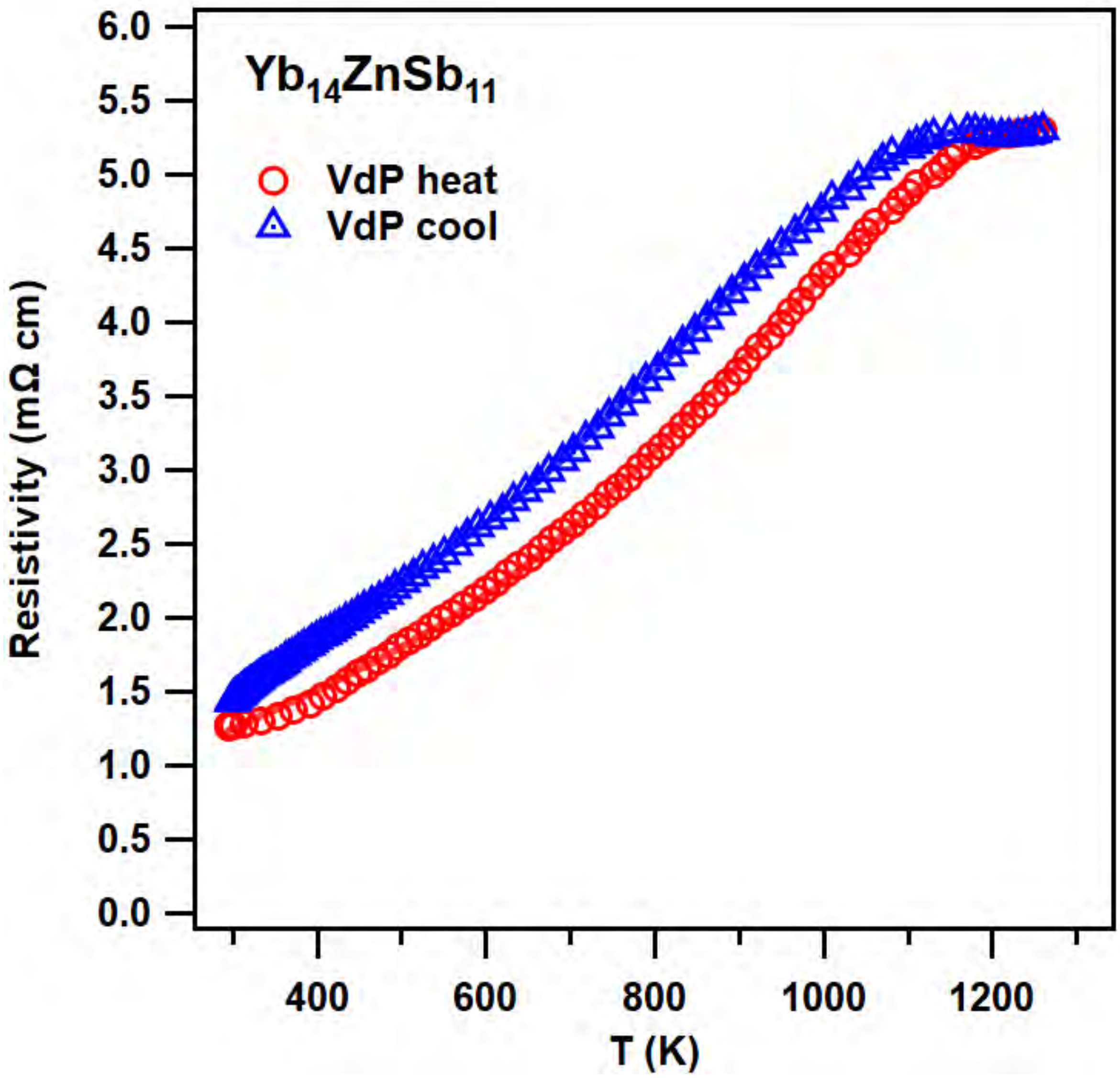

SI Figure S21. **Temperature dependent Van der Pauw electrical resistivity.** The electrical resistivity of $Yb_{14}ZnSb_{11}$ measured by the Van der Pauw method at JPL on heating (red, circles) and cooling (blue, triangles). The slight increase on cooling can be attributed to oxidation during the measurement.

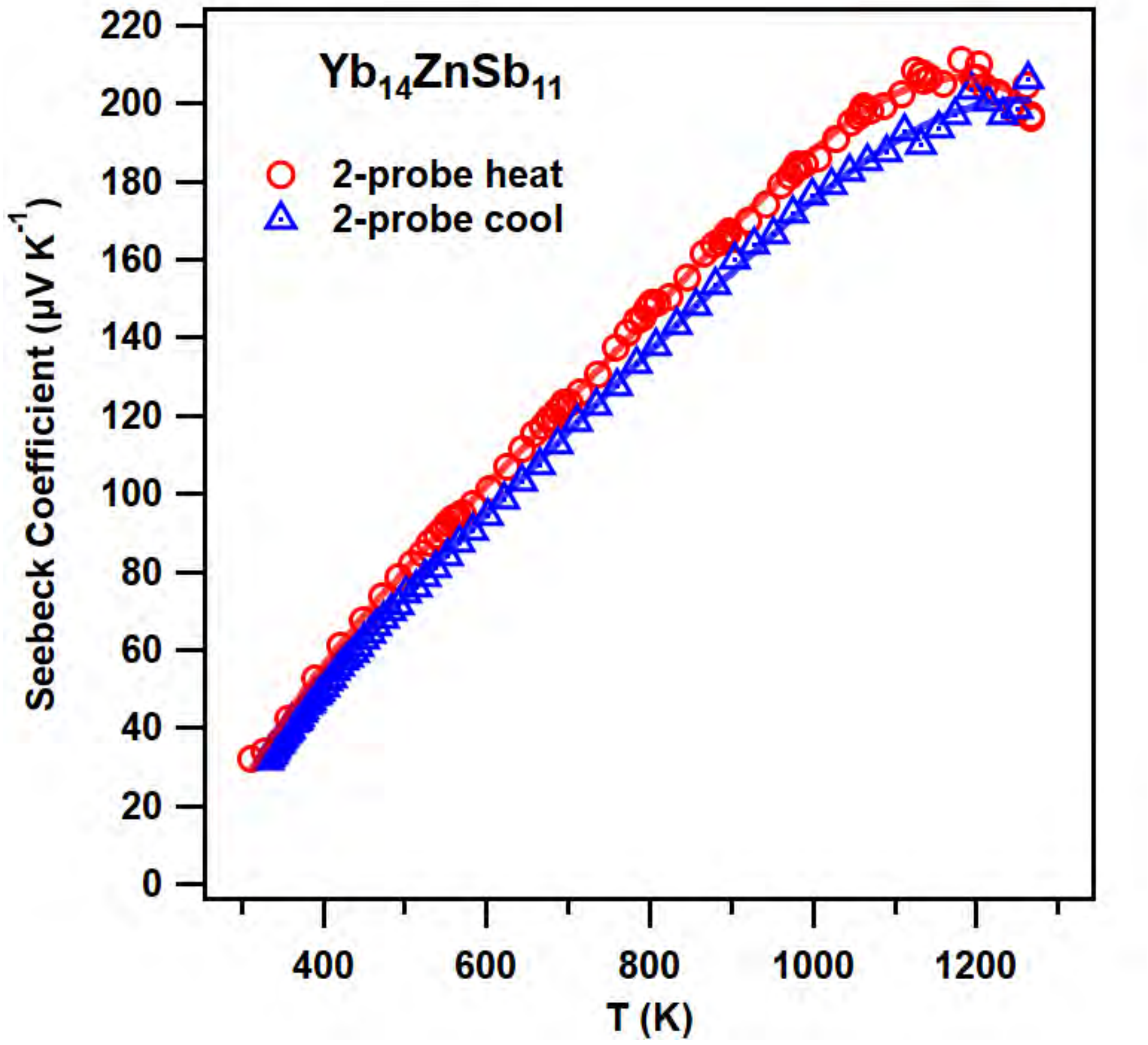

SI Figure S22. **Temperature dependent 2-probe Seebeck coefficients.** The Seebeck coefficient of $Yb_{14}ZnSb_{11}$ measured in a 2-probe orientation at JPL on heating (red, circles) and cooling (blue, triangles)

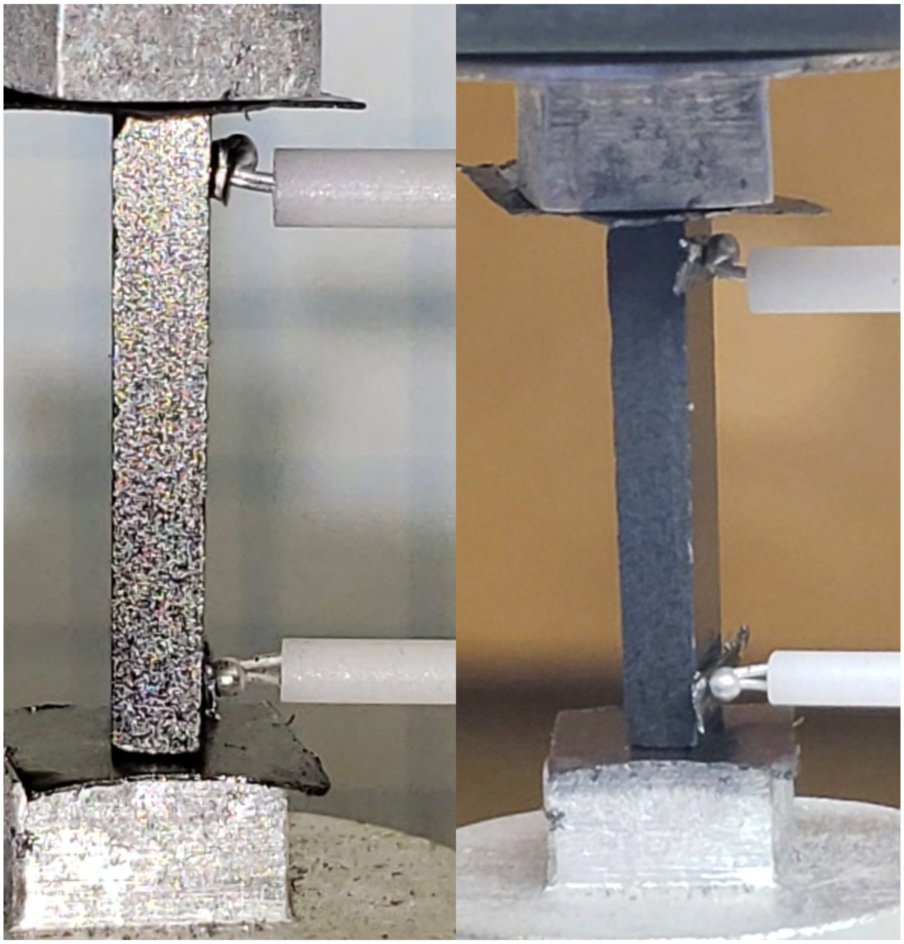

SI Figure S23. **Four probe Seebeck coefficient measurement on a commercial LSR-3 instrument.** A bar sample of $Yb_{14}ZnSb_{11}$ before (left) and after (right)**.** An 8 mm probe spacing was used on a 10 mm long bar and probes were place as close as possible to the ends of the bar to minimize the cold finger effect.

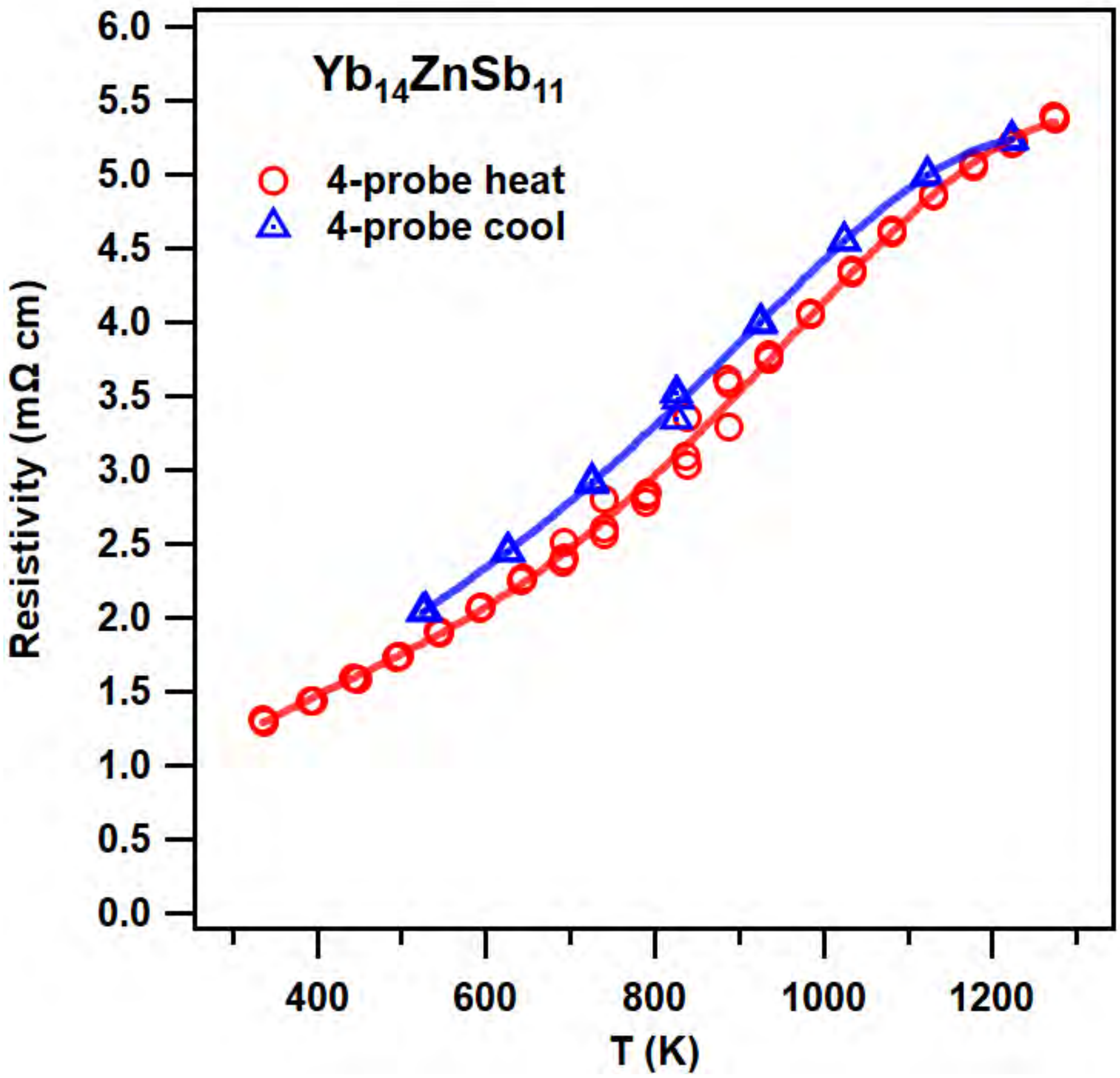

SI Figure S24. **4-probe temperature dependent electrical resistivity on a commercial LSR-3 instrument.** The temperature dependent electrical resistivity of $Yb_{14}ZnSb_{11}$. The heating is shown as red circles and the cooling are blue triangles. The slight increase can be attributed to oxidation.

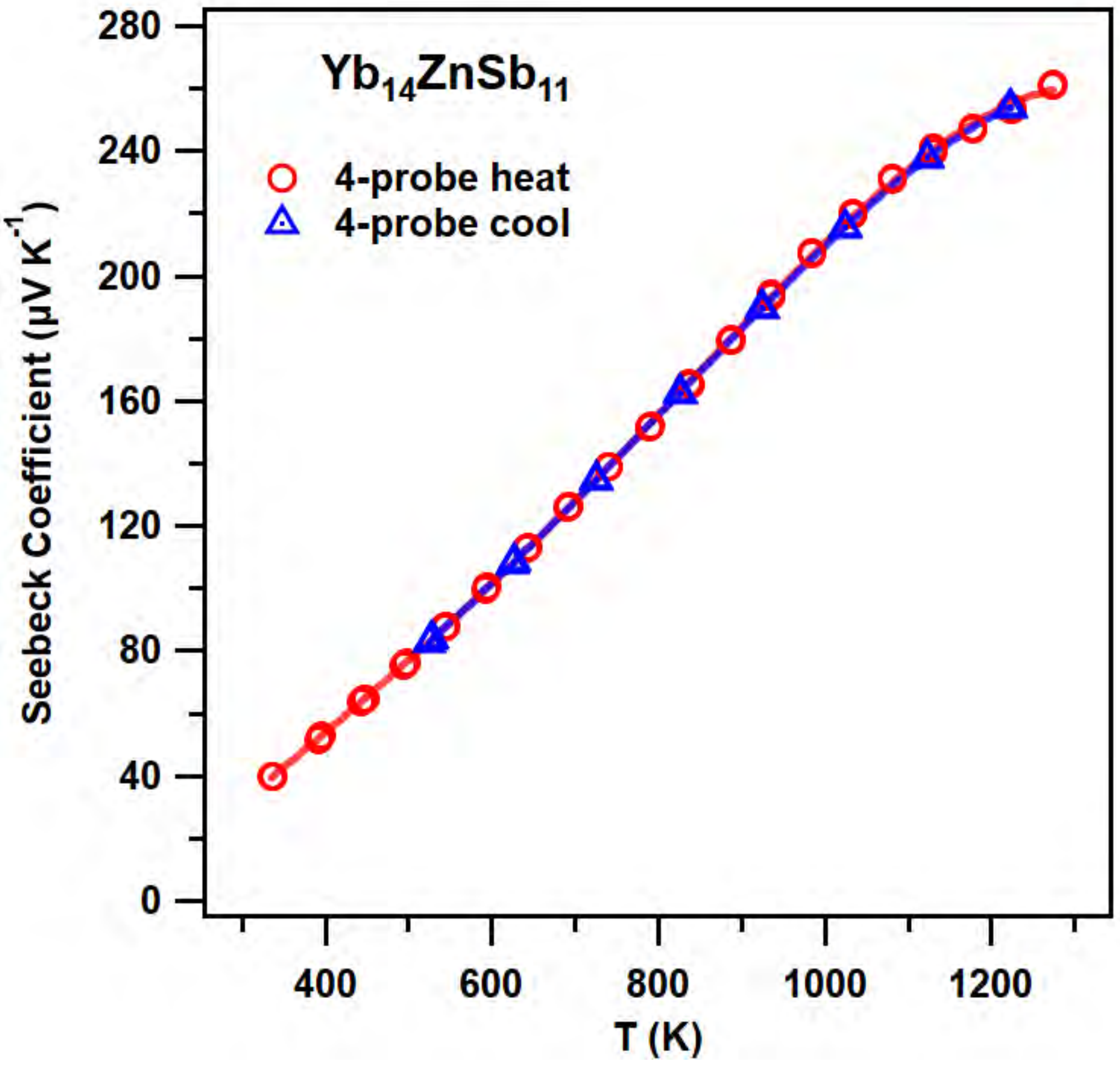

SI Figure S25. **Temperature dependent off axis 4-probe Seebeck measurement on a commercial LSR-3 instrument.** The temperature dependent Seebeck coefficient of $Yb_{14}ZnSb_{11}$ measured in a 4-probe orientation. The heating data are shown as red circles and the cooling data as blue triangles.
